# The cholesterol transporter Niemann-Pick C1 facilitates the entry of porcine epidemic diarrhea coronavirus

**DOI:** 10.1128/jvi.00301-26

**Published:** 2026-06-09

**Authors:** Siqi Li, Huaye Luo, Kang Zhang, Chuntao Wu, Yifeng Jiang, Yanjun Zhou, Changlong Liu

**Affiliations:** 1 Shanghai Veterinary Research Institute, Chinese Academy of Agricultural Sciences 118161 , Shanghai, China; 2 College of Animal Science and Technology, Guangxi University 622309, Nanning, China; 3 Office of Academic Research, Dongying Vocational Institute 829695, Dongying, China; 4 Jiangsu Co-Innovation Center for the Prevention and Control of Important Animal Infectious Disease and Zoonosis, Yangzhou University 38043, Yangzhou, People's Republic of China; University of Kentucky College of Medicine, Lexington, Kentucky, USA

**Keywords:** porcine epidemic diarrhea virus, cholesterol transporter, U18666A, Niemann-Pick C1, virus entry

## Abstract

Porcine epidemic diarrhea virus (PEDV) is an Alphacoronavirus that causes significant economic losses in the swine industry. It continues to pose a substantial threat to global swine production due to the limited efficacy of existing vaccines. Host-directed antiviral strategies targeting cellular pathways represent a promising approach to combat evolving viral strains. On the basis of our previous findings of a strong correlation between cholesterol transport and PEDV entry, we screened a panel of cholesterol-modulating compounds for anti-PEDV activity. We identified U18666A, an inhibitor of the Niemann-Pick C1 (NPC1) cholesterol transporter, as a potent and broad-spectrum inhibitor against multiple PEDV genotypes in both cell lines and porcine intestinal organoids. To validate NPC1 as a relevant pharmacological target, we utilized CRISPR/Cas9 to knock out NPC1 in Huh7 cells, which significantly inhibited PEDV infection. Conversely, forced expression of NPC1 in knockout cells partially restored viral infectivity. Using both authentic PEDV and PEDV pseudoviruses, we demonstrated that NPC1 facilitates the internalization of PEDV entry. Furthermore, co-immunoprecipitation and structural modeling revealed a direct interaction between NPC1 and the membrane-fusion subunit (S2) of the PEDV spike protein, as well as a role for NPC2 in this process, indicating that the NPC1/NPC2 cholesterol transport complex participates directly in viral entry. Additionally, exogenous cholesterol supplementation partially mitigated the inhibitory effect of U18666A, underscoring that functional intracellular cholesterol transport, not merely the presence of cholesterol, is required for efficient PEDV entry. Collectively, our findings elucidate a mechanism wherein PEDV co-opts the host NPC1/NPC2 cholesterol transport machinery for efficient internalization, thereby identifying this pathway as a promising target for the development of broad-spectrum host-directed antivirals.

**IMPORTANCE:**

Porcine epidemic diarrhea virus (PEDV) causes severe enteric disease in swine and continues to impose substantial economic losses on the global pig industry. Understanding the host determinants that govern PEDV entry is therefore critical for elucidating the mechanisms of infection. Here, we show that efficient PEDV entry is closely associated with intact and functional intracellular cholesterol transport in host cells. We identified the cholesterol transporter Niemann-Pick C1 (NPC1) as a critical host factor required for PEDV entry and demonstrated that NPC1 facilitates viral internalization through direct interaction with the S2 subunit of the PEDV spike protein. In addition, coordinated functions of the NPC1/NPC2 cholesterol transport system are involved in this process. By revealing the functional dependence of PEDV entry on host cholesterol trafficking, this study highlights this pathway as a potential target for therapeutic intervention.

## INTRODUCTION

Porcine epidemic diarrhea virus (PEDV), a member of the Alphacoronavirus genus within the Coronaviridae family, is the etiological agent of a highly contagious enteric disease in swine of all ages, posing a significant threat to global swine production (1). Since its first identification in the United Kingdom in 1971, PEDV has spread globally, causing severe enteric disease characterized by acute watery diarrhea and vomiting. The emergence of highly virulent strains in Asia around 2010, followed by their subsequent rapid global dissemination, caused substantial losses in the swine industry, with mortality rates approaching 100% in neonatal piglets (2, 3). Although commercial vaccines are available, their efficacy is limited by incomplete protection against emerging heterologous strains, including the spike (S) gene insertional (S-INDEL) variants. This situation underscores the necessity for novel antiviral strategies targeting host factors essential for viral replication, which may offer a higher genetic barrier to resistance (4).

PEDV encodes four major structural proteins, the spike (S), envelope (E), membrane (M), and nucleocapsid (N), as well as a variety of non-structural proteins essential for viral replication. The life cycle of coronaviruses, including PEDV, is initiated by the attachment and entry of the virion into a susceptible host cell, a process predominantly mediated by the viral S glycoprotein (5). The PEDV S protein is divided into S1 and S2 subunits; the S1 subunit facilitates attachment to host receptors, while the S2 subunit is responsible for driving the fusion of the viral and host membranes. Although several potential attachment factors and receptors have been proposed for PEDV, such as aminopeptidase N and sialic acids, the precise mechanisms governing its entry remain incompletely understood. This process appears to be complex and multifactorial (6–10). Following the binding to receptors, PEDV enters cells primarily through clathrin-mediated endocytosis. It then traffics through the endo-lysosomal pathway, where the low pH environment triggers further conformational changes in the S protein, ultimately culminating in viral genome release into the cytoplasm (11, 12). This intricate entry pathway is inherently reliant on a vast network of host cellular machinery, making these host factors attractive targets for broad-spectrum antiviral intervention.

Cellular cholesterol and lipid metabolism have emerged as critical facilitators for the replication of numerous enveloped viruses, including coronaviruses (13). Cholesterol is a vital structural component of lipid rafts-specialized, dynamic microdomains in the plasma membrane that serve as platforms for signal transduction and pathogen entry. Multiple steps of the coronavirus life cycle, from initial attachment and entry to replication complex formation and egress, are orchestrated within these cholesterol-rich domains (14–18). Specifically, for PEDV, previous studies have demonstrated a strong correlation between cellular cholesterol levels and viral infectivity, with the proposed mechanism being that the virus utilizes cholesterol to promote its entry (11, 19–21). Changes in membrane cholesterol levels, either drug-induced or caused by viral infection, may profoundly influence viral replication (22).

A pivotal component of the endo-lysosomal cholesterol transport machinery is the Niemann-Pick type C1 (NPC1) protein. NPC1 is a ubiquitously expressed, multi-pass transmembrane protein located in the limiting membrane of late endosomes and lysosomes (LE/Ly) (23, 24). Its essential function, in concert with its soluble partner NPC2, is to facilitate the efflux of unesterified cholesterol from the LE/Ly compartment to other cellular membranes, such as the endoplasmic reticulum and plasma membrane (25–27). In addition to its physiological role, NPC1 has been identified as an indispensable host factor for the entry of filoviruses, such as Ebola virus, serving as the intracellular receptor for the viral glycoprotein (28). Furthermore, growing evidence suggests that NPC1 is involved in the life cycles of other viruses, including some coronaviruses, although its role and necessity for PEDV infection have not been definitively established (29). In this study, we systematically screened a panel of cholesterol-modulating small molecules to identify potential inhibitors of PEDV infection. Among the compounds tested, U18666A emerged as a potent and broad-spectrum antiviral agent, demonstrating significant inhibition of PEDV across multiple genotypes. Our findings highlight U18666A as a promising cholesterol-modulating agent capable of broadly inhibiting PEDV infection. This study not only enhances our understanding of PEDV pathogenesis but also paves the way for the development of novel antiviral strategies targeting cholesterol-associated pathways in viral infections.

## MATERIALS AND METHODS

### Cells and virus

Vero cells (ATCC), HEK293T cells (ATCC, CRL-3216), Lenti-X 293T cells (Clontech), and Huh7 cells (provided by Prof. Rong Zhang, Fudan University) were cultured in Dulbecco’s modified Eagle’s medium (DMEM; Gibco) supplemented with 10% fetal bovine serum and 1% penicillin/streptomycin (Gibco) at 37°C in a humidified incubator with 5% CO₂. The following viruses were maintained in our laboratory: the PEDV SD strain (GenBank accession no. MZ596343) and PEDV HM strain (GenBank accession no. MZ342899); the recombinant viruses rPEDV-HM-EGFP and rPEDV-SD-EGFP, generated according to a previous protocol (30). rVSV-∆G-EGFP-G (VectorBuilder, Guangzhou, China) and the PEDV pseudovirus rVSV-∆G-EGFP-PEDV-S, which was prepared as described previously (31).

### Plasmid construction

The human codon-optimized, full-length spike gene of PEDV SD was synthesized by Saiheng Biotech (Shanghai, China) and inserted into the pLV-EF1a-IRES-Hygro vector. The S gene coding sequence, fused with a Strep tag at the C-terminus, was cloned and inserted into the pcDNA3.1(+) vector using EcoRI/XhoI sites. The coding sequences of both S1 and S2 genes containing the S protein signal peptide at the N-terminus and fused with an HA tag at the C-terminus were constructed into the pcDNA3.1 vector via the EcoRI/XhoI sites. Human codon-optimized NPC1 gene fused with a human IgG Fc tag and NPC2 gene fused with a Strep tag were synthesized by Gencefe Biotechnology (Wuxi, China) and inserted into the pLV-EF1a-IRES-Puro vector via the EcoRI/BamHI sites. The NPC1 A, C, and I domains were inserted into the pcDNA3.1 vector with an HA tag via the EcoRI/XhoI sites. sgRNAs targeting human NPC1 and NPC2 were designed, synthesized, and cloned and inserted into BsmBI-digested lentiCRISPRv2-Puro using T4 DNA ligase (NEB, Beijing, China). DNA oligonucleotide sequences used in this study are listed in Table S1.

### Chemical reagents and antibodies

U18666A (#HY-107433), LXR-632 (#HY-10629), Atorvastatin (#HY-B0589), and Evacetrapib (#HY-13327) were from MedChemExpress (MCE, Monmouth Junction, NJ, USA). Dalcetrapib (#CM03419) was purchased from Proteintech (Wuhan, China). Cholestyramine (#M9815) was from AbMole BioScience (Chengdu, Sichuan, China). Gemfibrozil (#S63853), Fenofibrate (#B65660), and Isoliquiritigenin (#B21525) were obtained from Yuanye (Shanghai, China). Water-soluble cholesterol (#C4951-30MG) was purchased from Sigma-Aldrich (St. Louis, MO, USA). Anti-β-tubulin (cat#: 66240-1-Ig), anti-GAPDH (cat#: 60004-1-Ig), and anti-Lamin B1 (cat#: 66095-1-Ig) antibodies were obtained from Proteintech. Anti-HA (cat#: 3724S) antibody was obtained from Cell Signaling Technology (Danvers, MA, USA). Anti-Strep II Tag (cat#: AE066) antibody was obtained from ABclonal. Anti-PEDV N (cat#: Ab-009-S) and anti-PEDV S (cat#: Ab-094) antibodies were purchased from QianXun Biotech (Guangzhou, China). Anti-NPC1 (cat#: EPR5209) and anti-NPC2 (cat#: EPR19993) antibodies were obtained from Abcam. HRP-conjugated anti-human IgG-Fc (cat#: 32935S) antibody was obtained from Cell Signaling Technology, and HRP-conjugated goat anti-rabbit and anti-mouse IgG (cat#: SA00001-1, SA00001-2) antibodies were purchased from Proteintech.

### Cell viability assay

Vero or Huh7 cells were seeded into 96-well plates at a density of 2,000 cells per well and allowed to adhere for 24 h. The cells were then exposed to the indicated compounds at a range of concentrations. Cell viability was assessed at 48 h post-treatment using a Cell Counting Kit-8 (MCE, Monmouth Junction, NJ, USA) following standard protocol. In brief, 10 µL of the CCK-8 solution was added to each well, followed by incubation at 37°C for 4 h. Absorbance was measured at 450 nm using a microplate reader (Synergy, USA). The half-maximal cytotoxic concentration (CC_50_) was calculated by nonlinear regression analysis using GraphPad Prism software (version 8.0.1).

### Lentivirus production

Lentivirus production was conducted as previously described (32). Briefly, Lenti-X 293T cells were co-transfected using calcium phosphate with the lentiviral vectors, the packaging plasmid psPAX2 (Addgene, Cat#: 12260), and the envelope-coding plasmid pMD2.G (Addgene, Cat#: 12259). Viral supernatants were harvested at 48 h post-infection (hpi), clarified by centrifugation at 2,000 rpm for 10 min, and filtered through a 0.45 μm cellulose acetate filter. The supernatants were then aliquoted and stored at −80°C for further use.

### Viral titration

Vero cells were plated into 96-well plates and cultured to 100% confluence. Viral samples were serially diluted 10-fold. For each dilution, eight replicate wells were inoculated with 100 µL of virus, and each sample was titrated in triplicate to ensure reproducibility. Following inoculation, cells were incubated for 7 days, and cytopathic effect (CPE) was recorded. Viral titers were calculated by the Reed-Muench method from the observed CPE and expressed as TCID_50_/mL.

### Flow cytometry analysis

Flow cytometric analysis was performed to determine the proportion of infected cells. Cells were dissociated with trypsin/EDTA (Gibco), harvested by centrifugation, and resuspended in 1 mL of FACS buffer consisting of PBS supplemented with 2 mM EDTA and 2% fetal bovine serum. GFP-expressing cells were analyzed using a NovoCyte Advanteon flow cytometer. For each sample, 10,000 events were collected. Data processing and analysis were carried out with FlowJo software (version 10; Ashland, OR, USA).

### Porcine intestinal organoid culture and PEDV infection

Porcine intestinal organoids were generated from the small intestinal tissues of 10-day-old piglets using established culture methods, as reported previously (33). Briefly, intestinal crypts were isolated, embedded in Matrigel, and dispensed as 50 μL domes into individual wells of 24-well plates. After Matrigel polymerization, organoid culture medium supplemented with 10 μM ROCK inhibitor was added, and organoids were maintained at 37°C in a humidified incubator with 5% CO₂.

To investigate the effect of U18666A on PEDV infection, viral infection experiments were performed using both two-dimensional (2D) and three-dimensional (3D) porcine intestinal organoid models. For the establishment of 2D cultures, 3D organoids were dissociated with TrypLE at 37°C for 15 min, and the resulting cells were seeded onto culture plates. Upon reaching confluence, cells were pretreated with U18666A (1 μg/mL) for 2 h and subsequently infected with rPEDV-HM-EGFP at a multiplicity of infection (MOI) of 0.1 for 2 h at 37°C. Following infection, the inoculum was removed, cells were washed, and fresh organoid culture medium was added for continued incubation for 24 h.

For infection of 3D organoids, Matrigel-embedded organoids were released using Cell Recovery Solution (Corning) by incubation on ice for 45 min. Organoids were collected in cold advanced DMEM/F12 and subjected to the same pretreatment and infection conditions as those used for the 2D system. After infection, organoids were re-embedded in Matrigel domes and cultured in organoid medium supplemented with U18666A throughout the culture period. Viral infection in both 2D and 3D organoids was examined at 24 hpi using fluorescence microscopy.

### Western blot

Cells were lysed on ice for 10 min in RIPA buffer (NCM Biotech, Suzhou, China) supplemented with phenylmethylsulfonyl fluoride (0.1 mM; Beyotime, Beijing, China). Cell lysates were combined with loading buffer and denatured either at 100°C for 10 min or at 37°C for 50 min for membrane protein analysis. Proteins were resolved via SDS-PAGE on 4%–12% precast gels (GenScript, Nanjing, China) and transferred onto nitrocellulose membranes (MerckMillipore, Billerica, MA, USA). Membranes were blocked with 5% nonfat milk for 2 h, followed by incubation with primary antibodies at room temperature for 1.5 h. After three washes with TBST containing 1% Tween-20, membranes were incubated with HRP-conjugated secondary antibodies (Proteintech, Wuhan, China) for 1 h at room temperature and washed again three times with TBST. Immunoreactive signals were visualized using an enhanced chemiluminescence detection kit (NCM Biotech, Suzhou, China) and captured with a ChemiDoc MP imaging system (Bio-Rad, Hercules, CA, USA).

### Co-immunoprecipitation

HEK293T cells were seeded in T25 flasks and co-transfected with the indicated plasmids at approximately 80% confluence using linear polyethylenimine (PEI, Noninbio, Shanghai, China). At 48 h post-transfection, cells were harvested and lysed on ice for 30 min in IP lysis buffer (Thermo Fisher Scientific, Waltham, MA, USA) supplemented with a protease inhibitor cocktail (Beyotime, Shanghai, China) and PMSF. Cell lysates were clarified by centrifugation, and a portion of the supernatant was retained as an input control. The remaining lysate supernatant was aliquoted for IP groups and incubated with Protein A/G agarose beads (Yeasen, Shanghai, China) or Strep-Tactin beads (Yuanye, Shanghai, China) at room temperature for 3 h with gentle rotation. Beads were washed five times with PBS containing 0.5% Tween-20, and bound proteins were eluted using elution buffer (Thermo Fisher Scientific, Waltham, MA, USA). Eluted samples were resolved by SDS-PAGE and subjected to western blot analysis to detect protein-protein interactions.

### Generation of NPC1 and NPC2 knockout cell lines

The sgRNA sequences targeting the NPC1 and NPC2 genes (Table S1) were designed and cloned into the LentiGuide-Puro vector using BsmBI restriction sites. Huh7 cells were transfected with the resulting sgRNA constructs using Lipofectamine 3000 (Thermo Fisher Scientific, Waltham, MA, USA). At 48 h after transfection, the cell culture was replaced with medium containing 2.5 µg/mL puromycin, and the cells were incubated for an additional 4 days. Monoclonal cell populations were subsequently obtained by limiting dilution in 96-well plates. Disruption of NPC1 expression in selected clones was confirmed by western blot analysis, genomic DNA sequencing, and PCR (Fig. S1).

NPC2 knockout cell lines were generated using a lentiviral CRISPR/Cas9 system. Briefly, lentiviral particles were produced in HEK293T cells and used to transduce Huh7 cells in the presence of 8 µg/mL polybrene. At 48 h post-transduction, cells were selected with 2.5 µg/mL puromycin for 4 days. Monoclonal cell populations were subsequently obtained by limiting dilution in 96-well plates. Loss of NPC2 protein expression in selected clones was confirmed by western blot analysis.

### RT-qPCR assay

Viral RNA was extracted from virus-infected cells using the VAMNE Magnetic Cell/Tissue Total RNA Kit (Vazyme, Nanjing, China). The cDNA was synthesized via reverse transcription using 5× PrimeScript RT Master Mix (Takara, Kusatsu, Japan). Quantitative real-time PCR was performed in triplicate using ChamQ Universal SYBR RT-qPCR Master Mix (Vazyme, Nanjing, China) following the manufacturer’s instructions for the detection of the PEDV nucleocapsid (N) gene. Relative expression was calculated using GAPDH as the reference gene. Primer sequences used for RT-qPCR are provided in Table S1.

### Virus attachment and internalization

Viral attachment and internalization assays were performed in NPC1 knockout cell lines. For viral attachment analysis, cells were incubated with rPEDV-SD-EGFP at an MOI of 10 for 2 h on ice to allow virus binding. Cells were then washed thoroughly with cold PBS to remove unbound virions, and cell-associated viral RNA was extracted and quantified by RT-qPCR. For virus internalization, infected cells (MOI = 10) were shifted to 37°C for 1 h and then treated with 1 mg/mL pronase (Solarbio, Beijing, China) to remove uninternalized virus. After three washes, cells were harvested for RNA extraction. Internalized viral RNA was quantified by RT-qPCR.

### Statistical analysis

Statistical analyses were performed using GraphPad Prism software (version 8.0.1; GraphPad Software, La Jolla, CA, USA). Data are presented as the mean ± standard deviation (SD) from at least three independent experiments. Comparisons between two groups were conducted using an unpaired two-tailed Student’s *t*-test, with statistical significance defined as *P* < 0.05 (*), *P* < 0.01 (**), and *P* < 0.001 (***).

## RESULTS

### Screening small-molecule compounds related to cholesterol as potential inhibitors of PEDV infection

**Fig 1:**
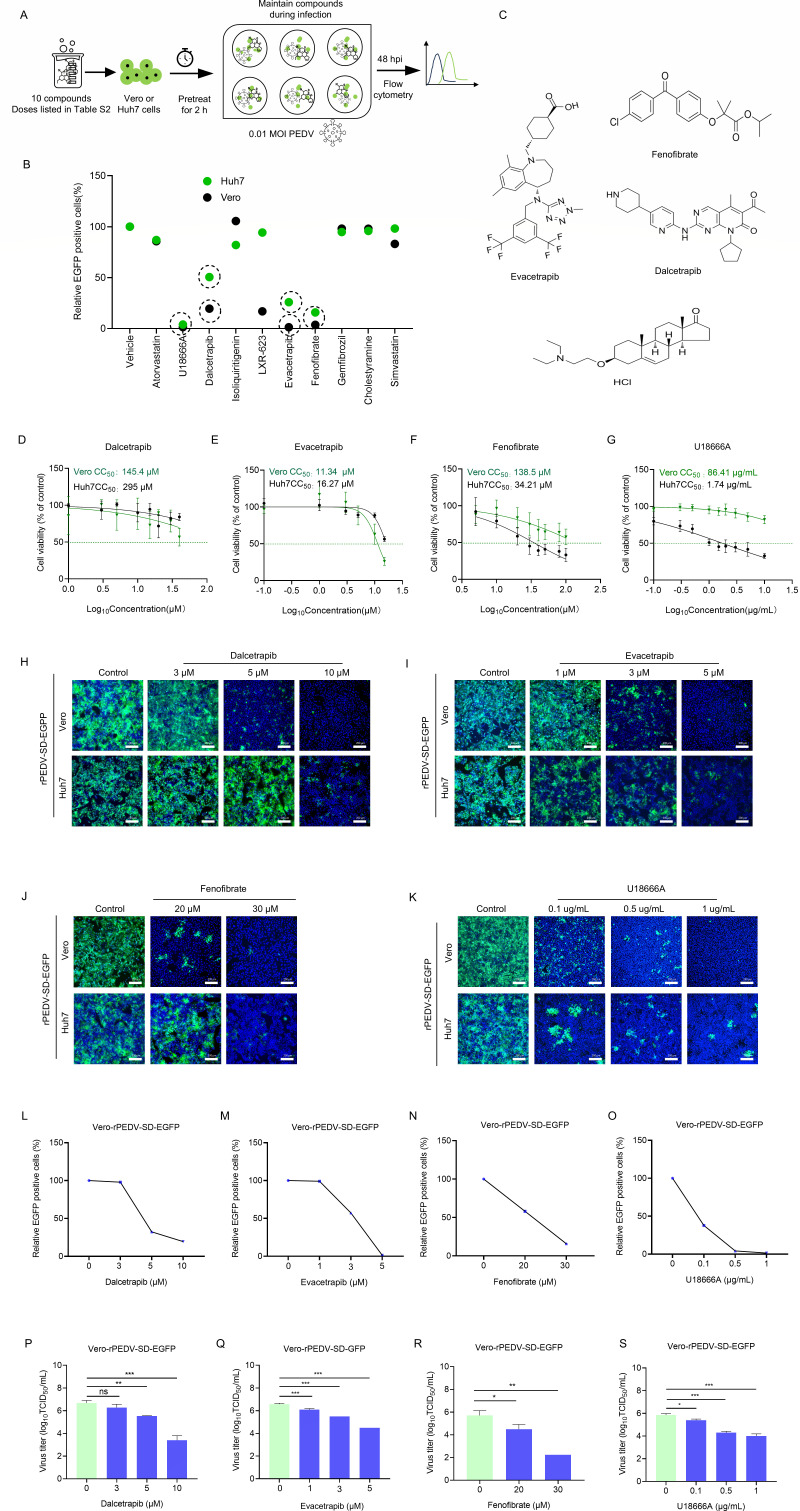
Cholesterol-based small-molecule compounds inhibit PEDV infection. (**A**) Schematic of the flow cytometry-based screening strategy for identifying PEDV inhibitors in Vero and Huh7 cells. (**B**) Antiviral activity of 10 candidate compounds against PEDV. Following a 2 h pretreatment with the indicated compounds, Vero (black) and Huh7 (green) cells were infected with rPEDV-SD-EGFP at an MOI of 0.01 in the continued presence of the compounds for 48 h. Infection was quantified by flow cytometry analysis of EGFP-positive cells. Data are presented as percentage inhibition relative to the no-drug control (set to 100% infection). (**C**) Chemical structures of four lead compounds (Dalcetrapib, Evacetrapib, Fenofibrate, and U18666A) identified in the screen are shown. (**D–G**) Cytotoxicity of the four compounds. Vero and Huh7 cells were treated with serially diluted compounds for 48 h, and cell viability was assessed using a Cell Counting Kit-8 (OD 450 nm). The 50% cytotoxic concentration (CC_50_) for each compound is indicated. (**H–K**) Representative fluorescence microscopy images of antiviral activity. Vero and Huh7 cells were infected with rPEDV-SD-EGFP (MOI = 0.01) and treated with the indicated concentrations of Dalcetrapib (**H**), Evacetrapib (**I**), Fenofibrate (**J**), or U18666A (**K**) for 48 h. Cell nuclei were stained with Hoechst 33342 (blue). Viral infection is indicated by the EGFP signal (green). Scale bars: 200 μm. (**L–O**) Quantitative analysis of infection by flow cytometry. The number of EGFP-positive cells was quantified, and the percentage of inhibition was calculated as in panel B. (**P–S**) Viral titers in culture supernatants. Infectious virus titers were determined by the TCID_50_ assay. Data are representative of three independent experiments and are presented as mean ± SD. Statistical significance was determined by one-way ANOVA with Dunnett’s test. ns: not significant; *: *P* < 0.05; **: *P* < 0.01; ***: *P* < 0.001.

To investigate whether host cholesterol pathways could serve as therapeutic targets against PEDV, we screened ten small-molecule inhibitors related to cholesterol transport and metabolism to identify compounds that could efficiently inhibit PEDV infection. A schematic diagram of the PEDV infection process is shown in Fig. 1A. To this end, Vero and Huh7 cells were pretreated with these compounds at the indicated concentrations (as listed in Table S2) for 2 h, then infected at an MOI of 0.01 with a recombinant PEDV (rPEDV-SD-EGFP) that expresses the EGFP marker and cultured in the presence of the compounds for 48 h. Using flow cytometry, we identified four compounds, including U18666A, Evacetrapib, Dalcetrapib, and Fenofibrate, that exhibited significant inhibitory activity against PEDV in both cell lines (Fig. 1B). Chemical structures of these four active compounds are presented in Fig. 1C. Among them, U18666A demonstrated the strongest inhibitory effect against PEDV (Fig. 1B). To ensure that the observed antiviral effects were not a consequence of cytotoxicity during compound treatment, we next determined the half-maximal cytotoxic concentration (CC_50_) values of these compounds in Vero and Huh7 cells using a cell viability assay. The result showed that the CC_50_ values of U18666A and Fenofibrate in Vero cells are much higher than those in Huh7 cells. In contrast, Dalcetrapib displayed a higher CC₅₀ value in Huh7 cells, whereas the CC_50_ values of Evacetrapib were similar in both cell lines (Fig. 1D through G). Thus, all subsequent experiments were conducted using non-cytotoxic concentrations of the compounds.

We further evaluated the antiviral activity of these four small molecules against PEDV infection at various concentrations in Vero and Huh7 cells. The results demonstrated that all four small molecules inhibited PEDV infection in a dose-dependent manner. This inhibition was confirmed by immunofluorescence microscopy (Fig. 1H through K), flow cytometry (Fig. 1L through O), and TCID₅₀ assay (Fig. 1P through S). Collectively, these findings demonstrate that inhibitors of cholesterol transport and metabolism effectively block PEDV entry in both Vero and Huh7 cells, with U18666A exhibiting the most potent antiviral activity.

### U18666A inhibits different genotypes of PEDV

**Fig 2:**
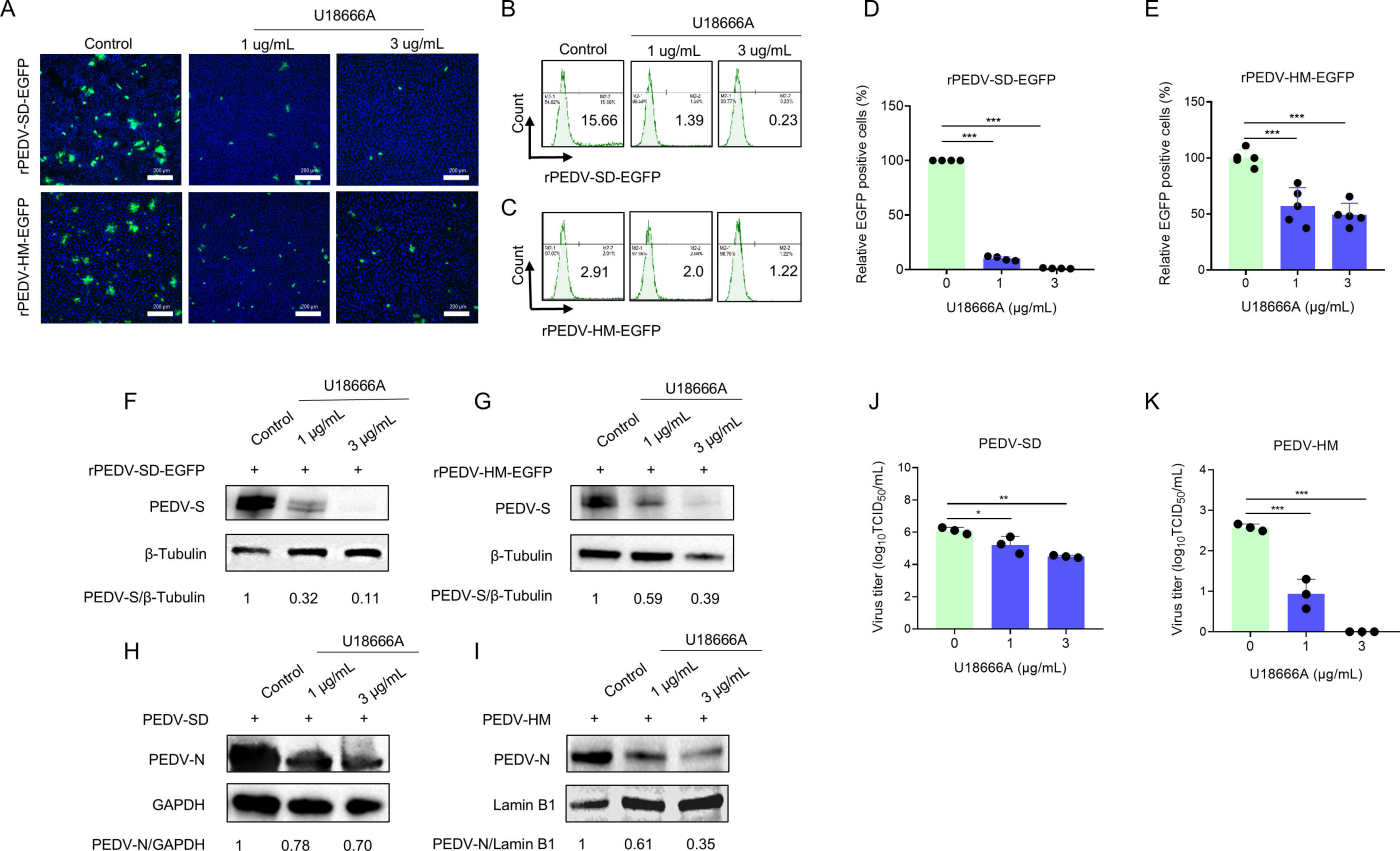
Effects of U18666A on different genotypes of PEDV strains. Vero cells were first pretreated with the indicated concentrations of U18666A (1 or 3 μg/mL) for 2 h. Subsequently, cells were infected with the indicated recombinant strains (rPEDV-SD-EGFP and rPEDV-HM-EGFP; MOI = 0.1) for 24 h in the presence of U18666A. (**A**) Representative fluorescence microscopy images showing S protein expression (green) via EGFP signal. Cell nuclei were stained with Hoechst 33342 (blue). Scale bars: 200 μm. (**B–C**) Quantification of viral infection by flow cytometry. The percentage of EGFP-positive cells was measured for each virus strain at the indicated U18666A concentrations. Representative flow cytometry plots are shown. (**D–E**) Quantitative analysis of the flow cytometry data from panels B and C, presented as the percentage of EGFP-positive cells for rPEDV-SD-EGFP (**D**) and rPEDV-HM-EGFP (**E**). (**F–I**) Western blot analysis of viral protein expression. Cell lysates were probed for the PEDV spike (S), protein (**F–G**), or nucleocapsid (N) protein (**H–I**). GAPDH, β-actin, or Lamin B1 served as loading controls. Band intensities were quantified using ImageJ software and normalized to the corresponding loading control. Data are presented as relative protein levels compared with the control group, which was set to 1. (**J**) Viral titers of PEDV SD and (**K**) HM strains in compound-treated supernatants were determined by the TCID_50_ assay. Data are representative of at least three independent experiments and are presented as mean ± SD (for panels D, E, J, and K). Statistical significance was determined by one-way ANOVA with Dunnett’s test. *: *P* < 0.05, **: *P* < 0.01, ***: *P* < 0.001.

To further investigate the antiviral efficacy of U18666A against PEDV, Vero cells were pretreated for 2 h with various concentrations of U18666A and then infected in the presence of U18666A for 24 h at an MOI of 0.1 with recombinant PEDV strains representing different genotypes: the G1 trypsin-independent strain SD and the G2 trypsin-dependent strain HM. The results from the EGFP fluorescence microscope assay (Fig. 2A) and flow cytometry confirmed that U18666A treatment led to a significant, dose-dependent reduction in the infection of both PEDV genotypes compared to the control group (Fig. 2B through E). Consistent with these findings, western blot analysis of the S protein demonstrated a similar trend, wherein a dose-dependent reduction in S protein expression was observed for all the tested PEDV (Fig. 2F and G).

To validate our findings with those of PEDV, we examined the effects of U18666A on PEDV strains SD and HM using the same cell pretreatment and infection protocol. Western blot analysis revealed dose-dependent reductions in PEDV N protein expression for both wild-type strains relative to the untreated control (Fig. 2H and I), and the TCID_50_ assay confirmed a significant decrease in progeny virion production, which was consistent with the reduced N protein levels (Fig. 2J and K). Collectively, these findings suggest that U18666A exhibits broad-spectrum antiviral activity against multiple genotypes of PEDV.

### U18666A inhibits PEDV infection in porcine intestinal organoids

**Fig 3:**
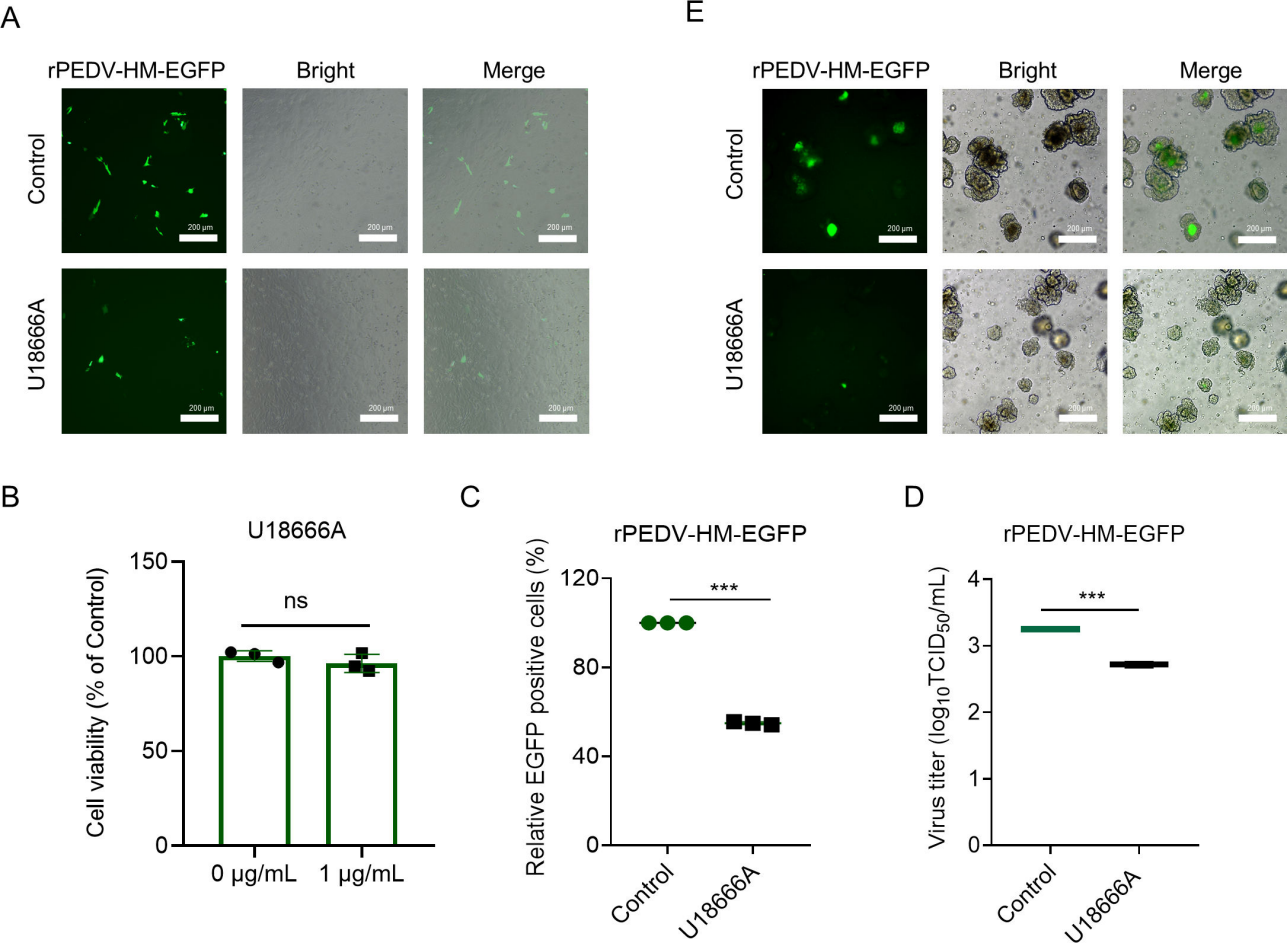
U18666A inhibits PEDV infection in both 2D and 3D porcine intestinal models. Porcine small intestinal crypts were isolated from neonatal piglets and cultured *in vitro* to establish both 2D organoids and 3D organoids. For infection, both models were pretreated with 1 µg/mL U18666A for 2 h, followed by infection with the recombinant strain rPEDV-HM-EGFP for 24 h in the continuous presence of U18666A. (**A**) Representative fluorescence microscopy images of 2D organoids. Scale bar: 200 µm. (**B**) Cell viability of 2D organoids after treatment with 1 µg/mL U18666A for 24 h was evaluated by CCK-8 assay. ns: not significant. (**C**) Quantitative analysis of infection rates in 2D organoids by flow cytometry. Data are presented as the percentage of EGFP-positive cells (mean ± SD; ***P < 0.001). (**D**) Viral titers in culture supernatants of 2D organoids at 24 hpi were determined by TCID_50_ assay. (**E**) Representative fluorescence microscopy images of 3D organoids. Scale bar: 200 µm.

To evaluate the antiviral efficacy of the drug U18666A in models that more closely mimic the *in vivo* physiological environment, we conducted a drug evaluation using porcine intestinal organoids. The organoids were isolated and cultured as previously described (33). For the initial assessment, we employed a 2D organoid culture system. Porcine intestinal organoids were dissociated into single cells, seeded onto culture plates, and pretreated with 1 μg/mL U18666A for 2 h. The cells were then infected with rPEDV-HM-EGFP at an MOI of 0.1, with U18666A maintained in the culture medium throughout the experiment. At 24 hpi, fluorescence microscopy, flow cytometry, and TCID₅₀ assays showed that U18666A significantly inhibited PEDV infection, whereas a CCK-8 assay demonstrated that 1 μg/mL U18666A exhibited no significant cytotoxicity under the tested conditions (Fig. 3A through D). To validate these findings, we applied the same treatment to 3D porcine intestinal organoids. The results revealed that U18666A retained significant antiviral activity at the same concentration (Fig. 3E). Collectively, these data indicate that U18666A possesses robust anti-PEDV activity in both organoid models, suggesting its potential as an antiviral candidate.

### U18666A effects on different infection steps of PEDV

**Fig 4:**
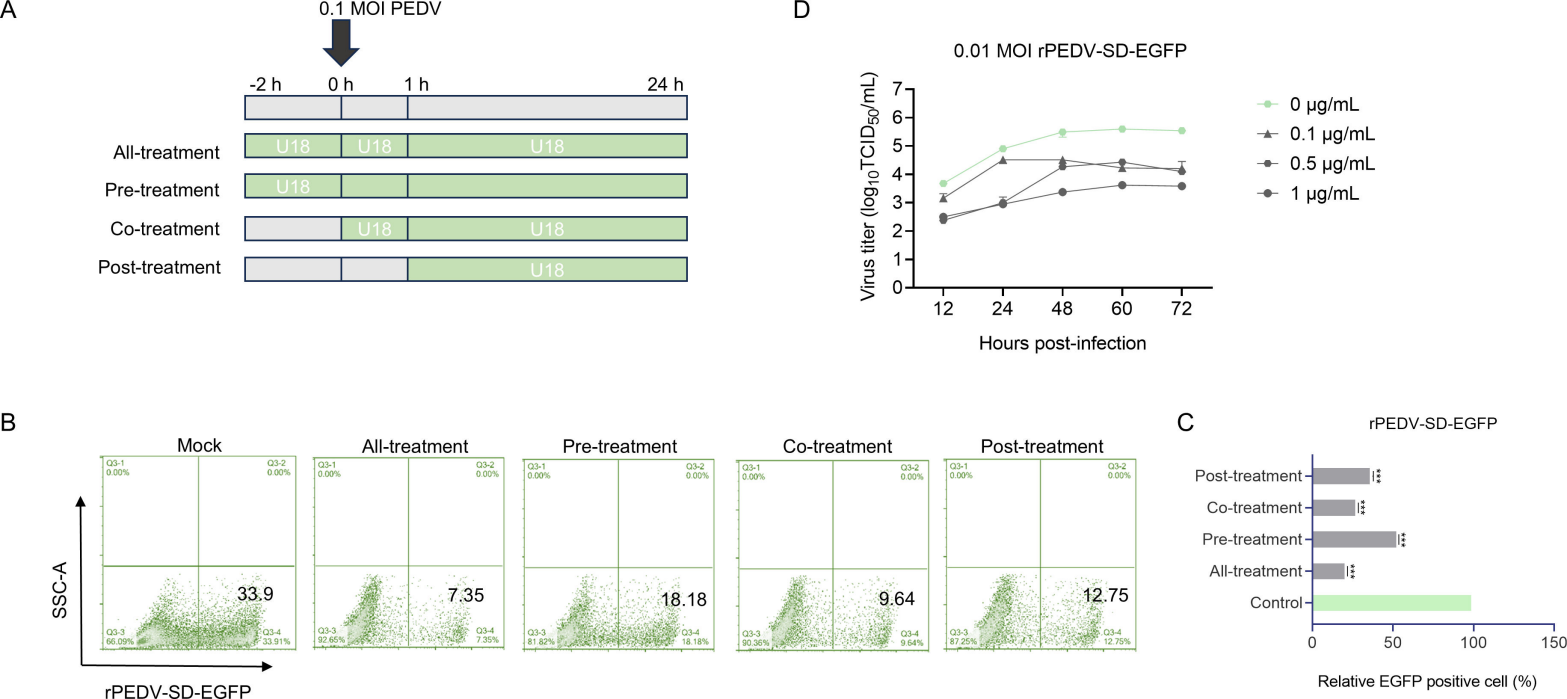
U18666A targets multiple stages of the viral life cycle and suppresses PEDV replication. (**A**) Schematics of the experimental timeline for U18666A treatment. Vero cells were treated with 3 μg/mL U18666A at the indicated time points relative to rPEDV-SD-EGFP. At 24 hpi, the cells were harvested and analyzed by flow cytometry. (**B**) Antiviral activity of U18666A against rPEDV-SD-EGFP. Viral infection was measured by EGFP-positive cells via flow cytometry. (**C**) Quantitative analysis of the EGFP-positive cell percentage from the experiment shown in panel B. Data are expressed as inhibition rate relative to the drug-free control (set to 100% infection). (**D**) Viral growth kinetics of PEDV in the presence of U18666A. Vero cells were infected with rPEDV-SD-EGFP (MOI = 0.01) and treated with the indicated concentrations of U18666A (0.1, 0.5, and 1 μg/mL) throughout the experiment. Culture supernatants were collected at 12, 24, 48, 60, and 72 hpi, and viral titers were determined by TCID_50_ assay. Data are representative of three independent experiments and presented as mean ± SD. Statistical significance was determined by one-way ANOVA with Dunnett’s test. ***: *P* < 0.001.

To investigate the stages of the PEDV life cycle targeted by U18666A, Vero cells were treated with U18666A and subsequently infected with rPEDV-SD-EGFP at an MOI of 0.1 under four regimens: all-treatment (before and after infection), pretreatment (4 h prior to infection), co-treatment (during virus infection), and post-treatment (administered 1 h post-infection) (Fig. 4A). The antiviral efficacy was evaluated at 24 hpi by flow cytometry. In the pretreatment, U18666A exhibited a significant antiviral effect, resulting in an approximately twofold reduction in viral infection. Both co-treatment and post-treatment with U18666A significantly diminished PEDV infection. Notably, the all-treatment condition yielded the most profound inhibitory effect (Fig. 4B and C).

We further evaluated the antiviral activity of U18666A by performing a viral growth kinetic assay. Vero cells infected with rPEDV-SD-EGFP (MOI = 0.01) were treated with U18666A at the indicated concentrations (0.1, 0.5, and 1 μg/mL). Supernatants were collected at 12, 24, 48, 60, and 72 hpi, and viral titers were determined by TCID_50_ assay. The results showed that U18666A inhibited PEDV replication in a dose-dependent manner. At 0.1 μg/mL, viral titers were initially comparable to the untreated group, but progressively diverged over time, as viral replication failed to reach the high levels observed in the control. In contrast, treatment with 0.5 and 1 μg/mL U18666A resulted in inhibition from early time points and maintained consistently low viral titers throughout the observation period (Fig. 4D). Collectively, these results demonstrate that U18666A exerts a robust and sustained antiviral effect against PEDV by targeting multiple stages of the viral life cycle in a dose-dependent manner.

### NPC1 is a critical factor for PEDV infection

**Fig 5:**
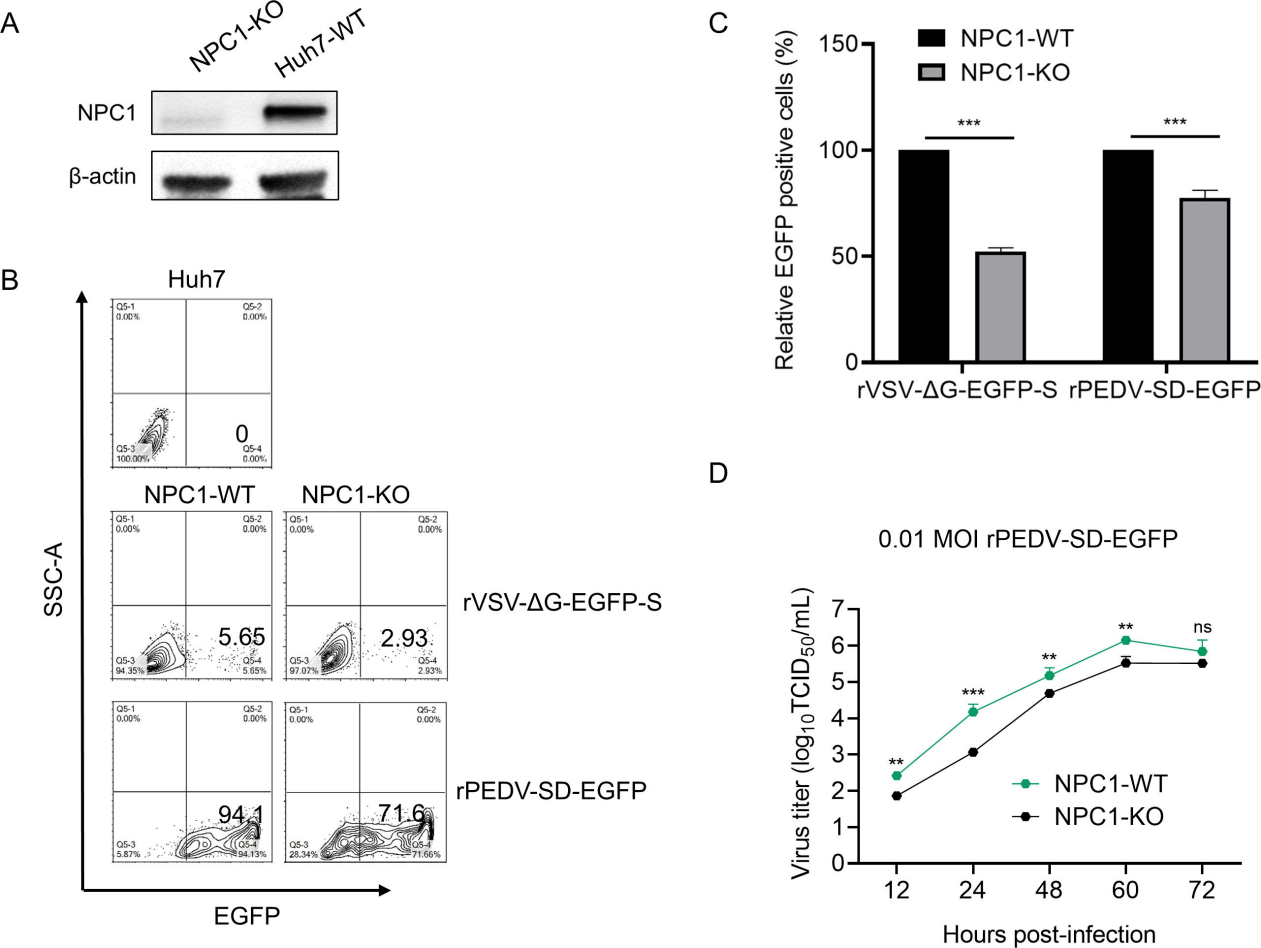
NPC1 is essential for PEDV infection. (**A**) NPC1 expression in sgRNA-transfected Huh7 cells was detected by western blot. (**B**) Flow cytometry analysis of PEDV infection in NPC1-WT and NPC1-KO Huh7 cells. Cells were infected with rVSV-ΔG-EGFP-PEDV-S or rPEDV-SD-EGFP at an MOI of 0.01. Samples were collected at 48 hpi, and infection efficiency was quantified by flow cytometry to measure the percentage of EGFP-positive cells. (**C**) Quantitative analysis of the EGFP-positive populations under each condition shown in panel B. (**D**) Viral growth kinetics analysis of PEDV in NPC1-WT and NPC1-KO Huh7 cells. Cells were infected with rPEDV-SD-EGFP at an MOI of 0.01. Culture supernatants were collected at 12, 24, 48, 60, and 72 hpi, and viral titers were determined by TCID_50_ assay. Data are from three independent experiments (mean ± SD; ns: not significant; **: *P* < 0.01; ***: *P* < 0.001).

U18666A is a small-molecule inhibitor that binds to and impairs the function of the NPC1 protein that blocks cholesterol export from late endosomes and lysosomes (34). Given that our previous results showed that U18666A effectively inhibits PEDV infection and that U18666A targets NPC1, we sought to examine the functional role of NPC1 in PEDV infection more specifically. To this end, we generated NPC1-knockout (NPC1-KO) Huh7 cell lines utilizing CRISPR/Cas9 gene editing. Single clones of NPC1-KO cells were selected and characterized by DNA sequencing, which confirmed frameshift deletions at the NPC1 gene locus (Fig. S1), and by western blot analysis, which demonstrated the absence of NPC1 protein expression in knockout clones (Fig. 5A). NPC1-KO cells and wild-type (WT) Huh7 cells were then infected with rVSV-ΔG-EGFP-PEDV-S or rPEDV-EGFP-SD at an MOI of 0.01 and then analyzed by flow cytometry at 48 hpi. The result revealed that NPC1-KO cells significantly inhibited PEDV infection compared to that in NPC1-WT cells (Fig. 5B and C). Viral growth kinetics assay further revealed that NPC1 deficiency delayed viral replication, with the NPC1-KO cells showing reduced viral titers, particularly at the early stages of infection (Fig. 5D). Taken together, these results suggest that NPC1 plays an important role in PEDV infection.

### NPC1 affects the internalization of PEDV

**Fig 6:**
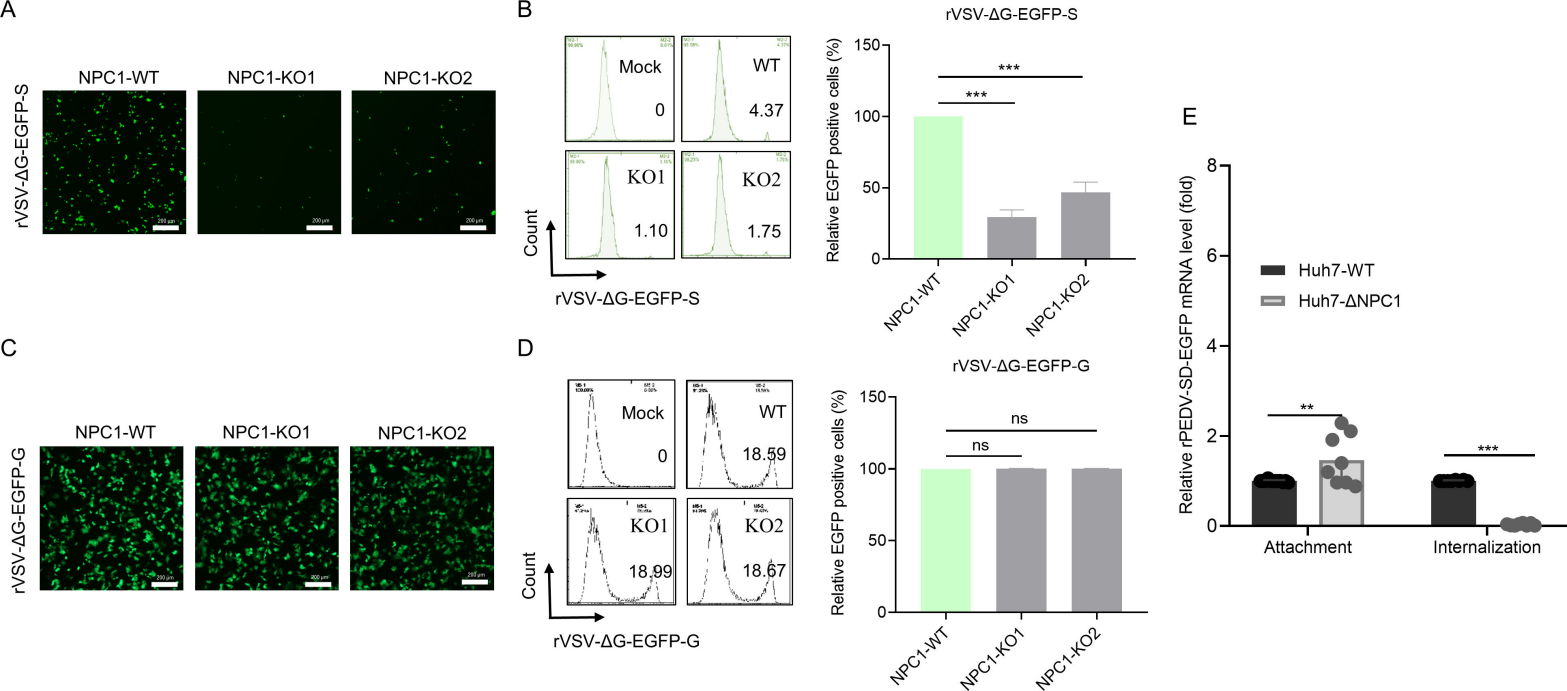
NPC1 is a critical factor for PEDV internalization. (**A**) NPC1-WT and NPC1-KO Huh7 cells were infected with the pseudotyped viruses rVSV-ΔG-EGFP-PEDV-S (MOI = 0.01) or rVSV-ΔG-EGFP-G (MOI = 0.1). Samples were collected at 24 hpi and analyzed via (**B**) flow cytometry and (**C**) fluorescence microscopy. Scale bars: 200 µm. (**D**) The relative transduction rates obtained from the flow cytometry analysis were quantified. (**E**) NPC1-WT and NPC1-KO Huh7 cells were infected with 10 MOI rPEDV-SD-EGFP for 2 h on ice. Cells were then washed with cold PBS three times, and viral attachment on the cell surface was detected by RT-qPCR. For internalization, after viral attachment for 1 h on ice, cells were shifted to 37°C for 1 h, washed, and intracellular RNA was analyzed. All data are shown as means ± standard errors of the means (*n* = 3 biological replicates). ns, not significant; **: *P* < 0.01; ***: *P* < 0.001.

Given that NPC1 may function as a host factor facilitating PEDV entry rather than merely serving as a restriction factor, we hypothesized that NPC1 plays an important role in enhancing the entry of PEDV into host cells. To test this hypothesis, we used a replication-deficient vesicular stomatitis virus (VSV) pseudovirus, rVSV-ΔG-EGFP-PEDV-S, in which the VSV glycoprotein gene was replaced with EGFP and the VSV glycoprotein was substituted with the PEDV spike protein (31). NPC1-WT and NPC1-KO cells were exposed to either rVSV-ΔG-EGFP-PEDV-S at an MOI of 0.01 or the control virus rVSV-ΔG-EGFP-G at an MOI of 0.1. Viral infection efficiency was quantified by fluorescence microscopy in combination with flow cytometric analysis. Compared with NPC1-WT cells, NPC1-KO cells exhibited a substantial decrease in rVSV-ΔG-EGFP-PEDV-S infection (Fig. 6A and B), but no appreciable difference was observed between the two cell types following infection with rVSV-ΔG-EGFP-G (Fig. 6C and D).

To further determine which step(s) of PEDV infection was affected by NPC1, we assessed viral attachment and internalization in NPC1-WT and NPC1-KO cells. For the attachment assay, NPC1-WT and NPC1-KO cells were incubated with PEDV (MOI = 10) on ice for 2 h to allow binding without internalization, then were washed with cold PBS. Cell-associated viral genomic RNA on the surface was measured by RT-qPCR. NPC1-KO cells showed stronger viral attachment than NPC1-WT cells (Fig. 6E). For internalization, virus-attached cells were shifted to 37°C for 1 h to permit uptake, and internalized viral RNA was detected by RT-qPCR. As shown in Fig. 6E, NPC1-KO cells exhibited significantly reduced internalization. Together, these results indicate that NPC1 facilitates PEDV internalization and thereby promotes viral entry.

### Forced expression of NPC1 partially restores PEDV infectivity in NPC1-KO cells

**Fig 7:**
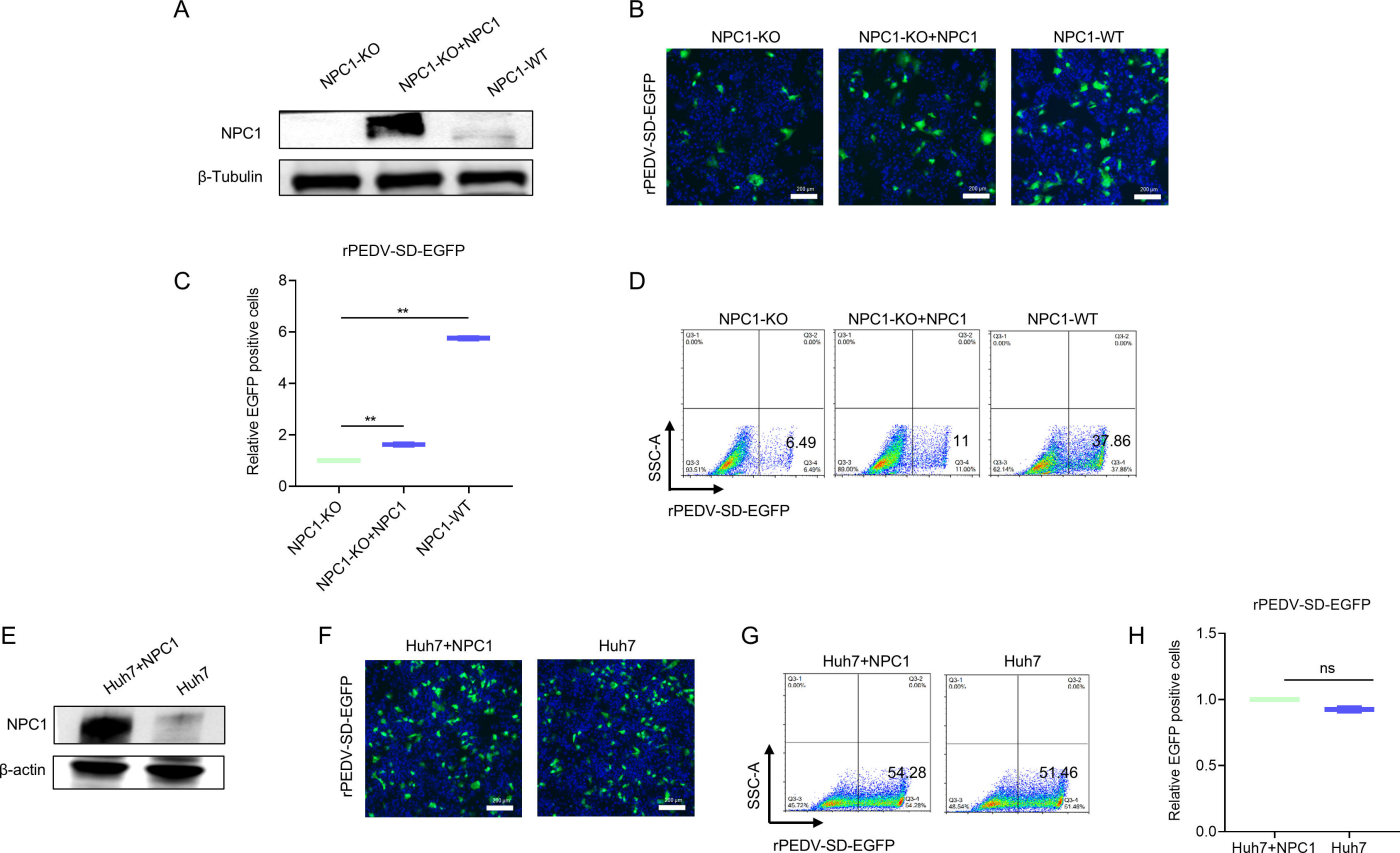
Exogenous NPC1 partially restores PEDV infectivity in NPC1 knockout cells. (**A**) Following the reintroduction of NPC1 into NPC1-KO cells via transduction with an NPC1-expressing or control empty vector, the cells were infected with rPEDV-SD-EGFP at an MOI of 0.1. Samples were collected at 24 hpi, and the level of the NPC1 protein was analyzed by western blot. (**B–D**) EGFP-positive cells were examined by fluorescence microscopy (scale bars: 200 µm) (B) and flow cytometry (C and D). (**E**) To further validate the role of NPC1, Huh7 cells were transfected with the NPC1-expressing or control vector. Following transfection, cells were infected and analyzed using the same approach as in panels A to D to assess viral infection. NPC1 protein levels were confirmed by western blot. (**F–H**) The EGFP-positive cells were imaged by fluorescence microscopy (scale bars: 200 µm) (F) or analyzed by flow cytometry (G and H). Data are presented as the mean ± SD of experiments. ns: not significant; **: *P* < 0.01.

To exclude the possibility that the phenotype observed in NPC1-deficient cells reflects off-target effects, we reintroduced NPC1 into NPC1-KO cells to test whether the viral infection efficiency could be restored. The transient transfection of NPC1 in NPC1-KO cells was confirmed through western blot analysis. The results showed that NPC1 was successfully expressed in the NPC1-KO cells, and notably, the expression of NPC1 in these cells was higher than the endogenous NPC1 levels observed in NPC1 wild-type (WT) cells (Fig. 7A). Then, the cells were infected with rPEDV-EGFP-SD (MOI = 0.1). At 24 hpi, the infection efficiency was quantified. The results indicated that the reintroduced NPC1 in NPC1-KO cells partially restored the capability for PEDV infection (Fig. 7B through D). Subsequently, we proceeded to overexpress NPC1 protein in NPC1-WT Huh7 cells. Interestingly, as shown in Fig. 7E through H, the overexpression of NPC1 protein in NPC1-WT Huh7 cells did not significantly affect the viral infection. These findings suggest that the endogenous levels of NPC1 are sufficient for facilitating PEDV infection.

### Exogenous cholesterol diminishes the inhibitory effect of U18666A on PEDV entry

**Fig 8:**
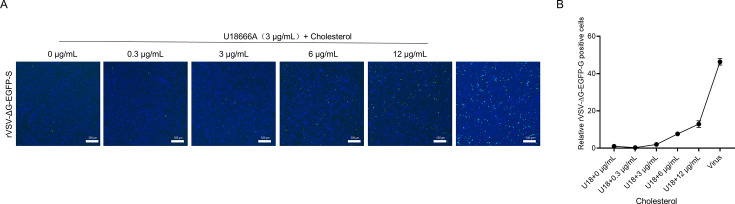
Exogenous cholesterol reverses U18666A-mediated blockade of viral entry. (**A**) Vero cells were pretreated with different concentrations of cholesterol for 12 h, followed by incubation with 3 μg/mL U18666A for 2 h. The compound was then removed prior to infection with 0.01 MOI of rVSV-ΔG-EGFP-PEDV-S for 12 h. Infection was then assessed by fluorescence microscopy and flow cytometry. Representative images show EGFP-positive cells (green) and Hoechst 33342-stained nuclei (blue). Scale bar: 500 μm. (**B**) To quantify the EGFP-positive cells observed in panel A, flow cytometry was performed on the same samples at 12 hpi. Data are presented as mean ± SD (*n* = 3).

Previous studies have shown that cholesterol promotes PEDV entry (19). However, whether this enhancement requires intact intracellular cholesterol trafficking remains unknown. To investigate this, Vero cells were treated with water-soluble cholesterol for 12 h, followed by the cholesterol transport inhibitor U18666A for 2 h. The compound was then removed, and cells were infected with rVSV-ΔG-EGFP-PEDV-S (MOI = 0.01). At 12 hpi, the infection efficiency was quantified as the percentage of EGFP-positive cells by flow cytometry. The result demonstrated that exogenous cholesterol partially restored PEDV pseudovirus infection in U18666A-treated cells, as evidenced by a significant increase in EGFP-positive cells (Fig. 8A and B). However, infection did not reach the level observed in untreated normal cells, suggesting that the efficient entry of PEDV requires intact intracellular cholesterol trafficking.

### NPC1 interacts with PEDV S protein, specifically the S2 subunit, but not the S1 subunit

**Fig 9:**
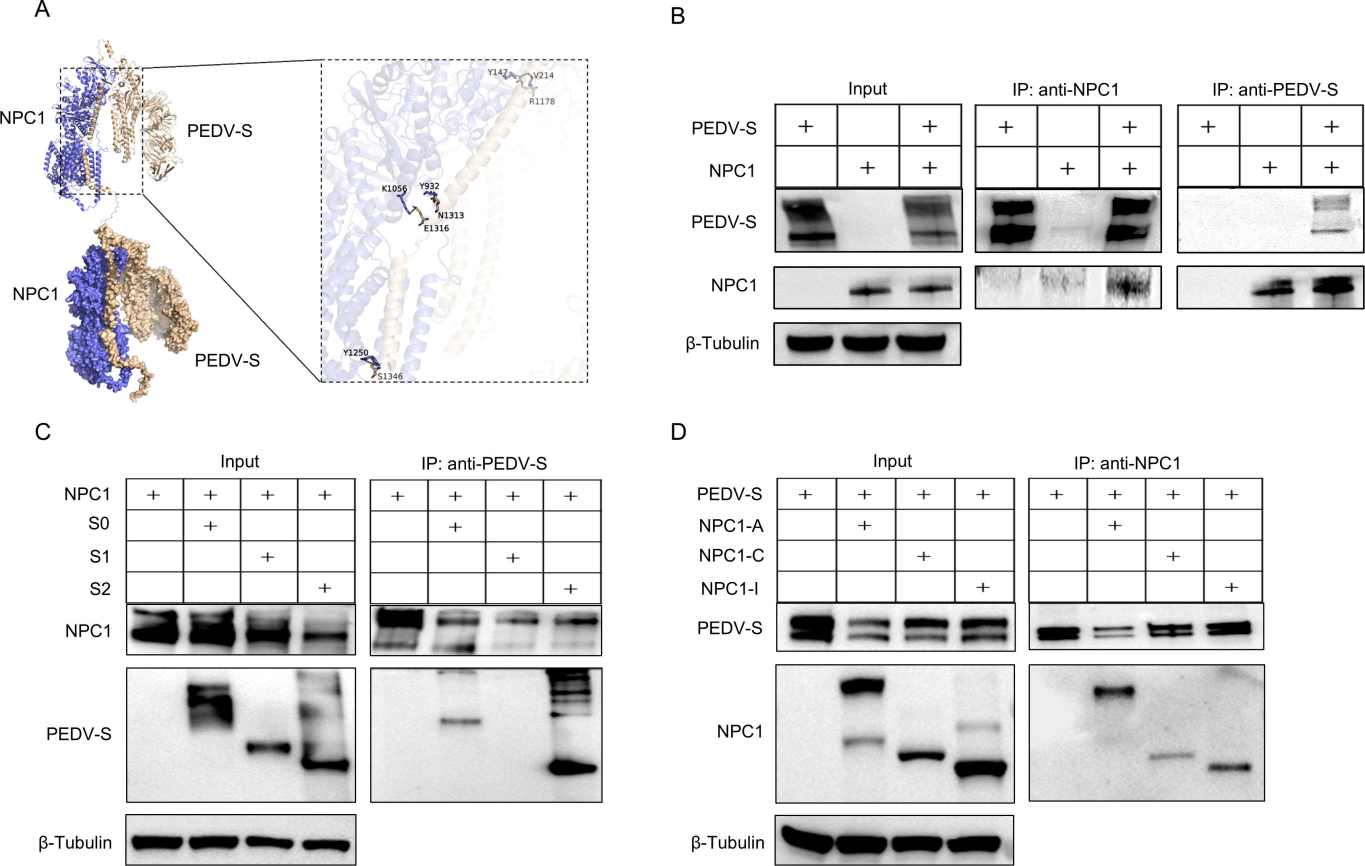
NPC1 interacts with PEDV S protein. (**A**) Predicted structural model of the NPC1 and PEDV S protein complex. The complex was modeled using the AlphaFold Server, and the top-ranking prediction was evaluated for binding affinity using Prodigy (35). The conformation with the most favorable energy is shown, visualized in PyMOL 2.5.3. PEDV S structure is shown in yellow, while the NPC1 is depicted in purple. The yellow dotted line in the enlarged picture indicates hydrogen bonding. (**B**) Co-IP analysis of the interaction between NPC1 and PEDV S. HEK293T cells were co-transfected with plasmids encoding NPC1 and the PEDV S. Proteins from cell lysates (input) and immunoprecipitated (IP) were detected by western blot. (**C**) NPC1 interacts with PEDV S2. PEDV S0, S1, or S2 were expressed with NPC1 in HEK293T cells. These S proteins were immunoprecipitated, and the proteins were detected by western blot. (**D**) NPC1 domains A, C, and I are capable of binding to PEDV S. The three domains of NPC1 (domain A: 1–270 aa; domain C: 380–625 aa; domain I: 854–1098 aa) were co-expressed with full-length PEDV S in HEK293T cells. After immunoprecipitation of the NPC1 domains, their association with PEDV S was assessed by western blot.

To investigate the potential interaction between NPC1 and the PEDV S protein, we first employed an integrated structural prediction approach. Structural models of the NPC1 and PEDV S proteins were generated using AlphaFold and subsequently assessed for their ability to form a complex. The binding energy of the top-ranked predicted NPC1-PEDV S protein complex exhibited was −14.3 kcal/mol, as determined by the Prodigy server. The protein-protein binding affinity reflected by the docking score, as shown in Fig. S2, indicates a strong binding interaction. Visualization of the predicted complex using PyMOL revealed a prominent network of hydrogen bonds at the interface between NPC1 and PEDV S protein (Fig. 9A), suggesting that hydrogen bonding constitutes the primary mode of interaction. Given the established role of hydrogen bonding in protein-protein electrostatic complementarity, these findings imply a highly stable and specific association between NPC1 and the PEDV S protein. To further validate the interaction between NPC1 and PEDV S protein, we performed a co-immunoprecipitation (Co-IP) assay in HEK293T cells that were co-transfected with expression plasmids for NPC1 and PEDV S protein. The result demonstrated the co-precipitation of NPC1 with the PEDV S protein (Fig. 9B). In a parallel experiment, a Strep-tag II pull-down assay further confirmed the association between the two proteins (Fig. 9B).

The PEDV S protein is functionally divided into the receptor-binding S1 subunit and the membrane-fusion S2 subunit. To delineate the specific region of the S protein that interacts with the NPC1 protein, we constructed truncated expression vectors for both the S1 and S2 subunits. These vectors were co-transfected into cells alongside NPC1 expression vectors, followed by a Co-IP assay to examine protein-protein interactions. Our results revealed a specific association between NPC1 and the S2 subunit of the PEDV S protein, with no detectable interaction with the S1 subunit (Fig. 9C). We next sought to identify the NPC1 domains that are pivotal for binding to the PEDV S protein. We assessed interactions with three distinct domains of NPC1: domain A (1–270 aa), domain C (380–625 aa), and domain I (854–1098 aa). Co-IP assays demonstrated that the S protein engages with all three domains. However, the interactions varied in strength. Notably, robust binding was observed with domain A and domain I, while a weaker interaction was noted with domain C (Fig. 9D). Collectively, these findings are corroborated by computational modeling and highlight the specific binding interactions between NPC1 and the PEDV S protein. These findings emphasize the crucial role of the S2 subunit and NPC1 in the viral entry mechanism.

### NPC2 cooperates with NPC1 to facilitate PEDV infection

**Fig 10:**
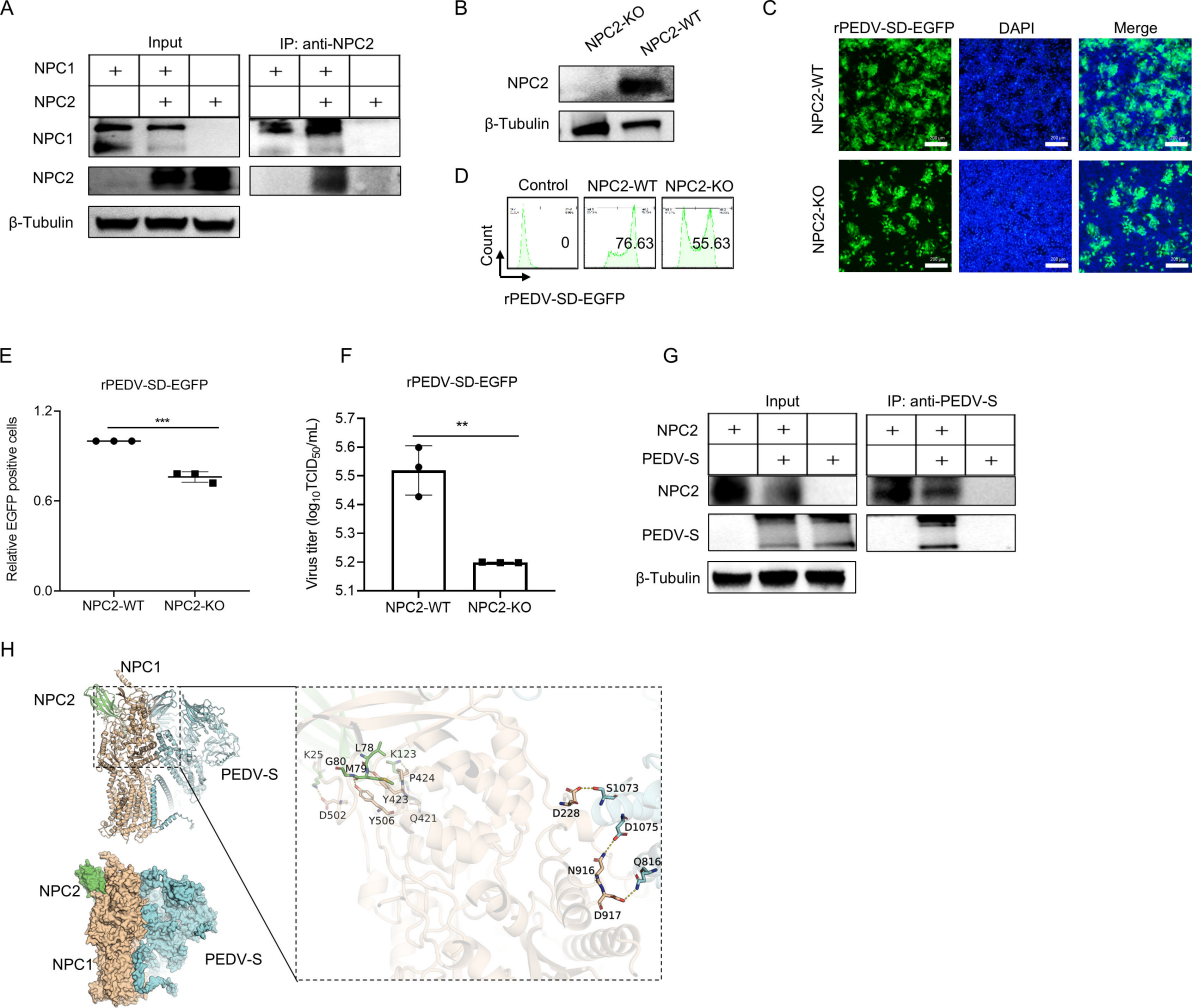
NPC2 and its assembly with NPC1 into a ternary complex are essential for PEDV infection. (**A**) Co-IP confirms the interaction between NPC1 and NPC2. Proteins were co-expressed in HEK293T cells, and their association was assessed by Co-IP, followed by western blot. (**B**) Generation of NPC2-knockout (NPC2-KO) Huh7 cells. Huh7 cells were transduced with sgRNAs targeting NPC2, and knockout efficiency was confirmed by western blot. (**C**) NPC2 deficiency impairs PEDV infection. NPC2-KO and NPC2-WT cells were infected with 0.01 MOI of rPEDV-SD-EGFP for 24 h. Viral infection was visualized by fluorescence microscopy, showing EGFP-positive cells (green) and Hoechst 33342-stained nuclei (blue). Scale bars: 200 μm. (**D**) Flow cytometry analysis of EGFP-positive cells in panel C. (**E**) Quantitative analysis of EGFP-positive cells in panel D. (**F**) Viral titers in the culture supernatants in panel C were determined by TCID_50_ assay. Data are presented as mean ± SD (*n* = 3). **: *P* < 0.01; ***: *P* < 0.001. (**G**) NPC2 interacts with the PEDV S protein. Co-IP assays were performed in HEK293T cells co-expressing NPC2 and PEDV S protein. (**H**) Predicted structural model of the NPC1–NPC2–PEDV S ternary complex. The computational model depicts NPC1 (yellow), NPC2 (green), and PEDV S (blue). The inset provides a detailed view of the interaction interface, with yellow dashed lines representing predicted hydrogen bonds that stabilize the complex.

It has been reported that NPC1 and NPC2 cooperatively mediate cholesterol egress from late endosomes (28, 36). We initially confirmed their interaction through a co-immunoprecipitation assay (Fig. 10A). Given our prior finding that NPC1 promotes PEDV infection, we hypothesized that NPC2 is also required for PEDV infection. To test this hypothesis, we generated NPC2-knockout (NPC2-KO) Huh7 cells using CRISPR/Cas9 (Fig. 10B) and subsequently challenged them with rPEDV-SD-EGFP at an MOI of 0.01. NPC2-KO cells exhibited a significant reduction in PEDV infection compared to the wild-type control (NPC2-WT), as evidenced by diminished EGFP signals observed through fluorescence microscopy and flow cytometry (Fig. 10C through E). Consistently, viral titers in the culture supernatants were markedly lower in NPC2-KO cells than in NPC2-WT cells (Fig. 10F), further confirming the critical role of NPC2 in efficient PEDV infection.

We next investigated whether NPC2 interacts with the S protein. Co-IP experiments demonstrated that NPC2 was associated with PEDV S (Fig. 10G). Together with the NPC1–NPC2 interaction, these findings support the existence of a multicomponent complex comprising NPC1, NPC2, and PEDV S at the host-virus interface. To gain structural insight into this assembly and to mechanistically elucidate the interaction data, we performed molecular docking to model the ternary complex. The predicted architecture positions NPC1 as a central scaffold, with its left domain engaging NPC2 and its right domain associating with PEDV S, thereby yielding a stable tripartite complex (Fig. 10H). This organization is consistent with a model in which PEDV utilizes the NPC1/NPC2-dependent cholesterol transport machinery to facilitate infection.

## DISCUSSION

PEDV remains a significant threat to global swine production (37). Currently available vaccines cannot completely protect pigs from PEDV infection. This underscores the urgent need for novel antiviral strategies targeting host factors critical for viral replication. Our previous transcriptomic analysis revealed a strong correlation between cholesterol homeostasis and PEDV entry, identifying ezetimibe as an effective inhibitor (19). Based on this, we screened a panel of cholesterol-modulating compounds and identified U18666A, Evacetrapib, Dalcetrapib, and Fenofibrate as potent, dose-dependent inhibitors of PEDV infection. Among these, U18666A emerged as a potent inhibitor of PEDV infection, showing antiviral activity against multiple PEDV genotypes in cell lines and against the PEDV HM strain in porcine intestinal organoids. Given that U18666A targets NPC1, we further demonstrated that the NPC1 protein is an essential host factor for PEDV entry. In addition, NPC1, in concert with its partner NPC2, facilitates viral internalization through direct interaction with the PEDV spike protein, revealing a previously unrecognized entry mechanism for this coronavirus.

Cholesterol, as a core component of plasma membranes and lipid rafts, is known to facilitate various steps in the viral life cycle (38–40). Viruses cross membrane barriers and ultimately exit via membrane-enclosed compartments (41, 42). This process is highly dependent on the specific interaction between membrane lipids (especially cholesterol) and viral proteins (43). In the context of coronaviruses, cholesterol-rich domains have been implicated in virus entry (44); however, the specific role of NPC1 has remained ambiguous. To address this gap, our data provide compelling evidence that PEDV directly co-opts the NPC1 pathway, revealing a mechanism that is distinct from those employed by other viruses. While NPC1’s role in Ebola virus entry involves binding the cleaved glycoprotein to trigger endosomal escape (45), its function in SARS-CoV-2 may be partially independent of its cholesterol transport (29, 46). Our findings suggest that PEDV hijacks the entire NPC1/NPC2 cholesterol transport machinery. Importantly, we observed that the interaction between NPC1 and the PEDV spike protein is specific to the S2 subunit, with no detectable binding to the S1 subunit. Since the S1 subunit mediates initial receptor attachment and the S2 subunit facilitates membrane fusion, this S2-specific interaction positions NPC1’s function at a post-attachment step, likely aiding the conformational changes in S2 required for fusion. This is consistent with our functional data showing that NPC1 deficiency impairs internalization but not initial attachment, indicating that NPC1 contributes both to efficient uptake and to subsequent fusion steps. Given the increased viral attachment observed in NPC1-KO cells, we hypothesize that this phenomenon is due to impaired downstream internalization, which could prolong the retention of virions on cell-surface attachment factors, such as sialic acids, and thereby contribute to the increased attachment signal (47–49). This mechanism, occurring after the virus has been internalized and trafficked to the late endosome, could explain the broad-spectrum inhibitory effect of U18666A across PEDV genotypes, given the higher conservation of the S2 subunit. It should be noted that, in addition to disrupting cholesterol transport and NPC1-associated functions, U18666A treatment may also partially inhibit PEDV entry through effects on endosomal acidification and/or protease activity. Because PEDV entry relies on low pH and endosomal proteases, this potential mechanism must be considered (50). Although the effect of U18666A on lysosomal/endolysosomal pH remains controversial in the literature (51), our cholesterol replenishment experiment and the reduced susceptibility of NPC1-deficient cells more strongly support disruption of NPC1-dependent cholesterol trafficking as the predominant mechanism underlying U18666A-mediated inhibition of PEDV entry.

Our findings are further supported by recent discoveries. A study by Khan et al. identified natural compounds that inhibit coronavirus and filovirus entry by directly targeting NPC1 (29). They demonstrated that the luminal domain C of NPC1 binds directly to the receptor-binding domain of the SARS-CoV-2 spike, which converges with our model by establishing NPC1 as an authentic intracellular receptor for coronaviruses. Our observation that NPC1 ablation blocks internalization aligns with the model (52), which demonstrated that NPC1 inhibition depletes plasma membrane cholesterol, thereby impairing clathrin-coated pit dynamics and preventing vesicle scission. This offers a unifying mechanism for the broad-spectrum antiviral activity of NPC1 inhibitors: by disrupting cholesterol trafficking, these inhibitors impair a fundamental cellular portal utilized by various unrelated viruses.

**Fig 11:**
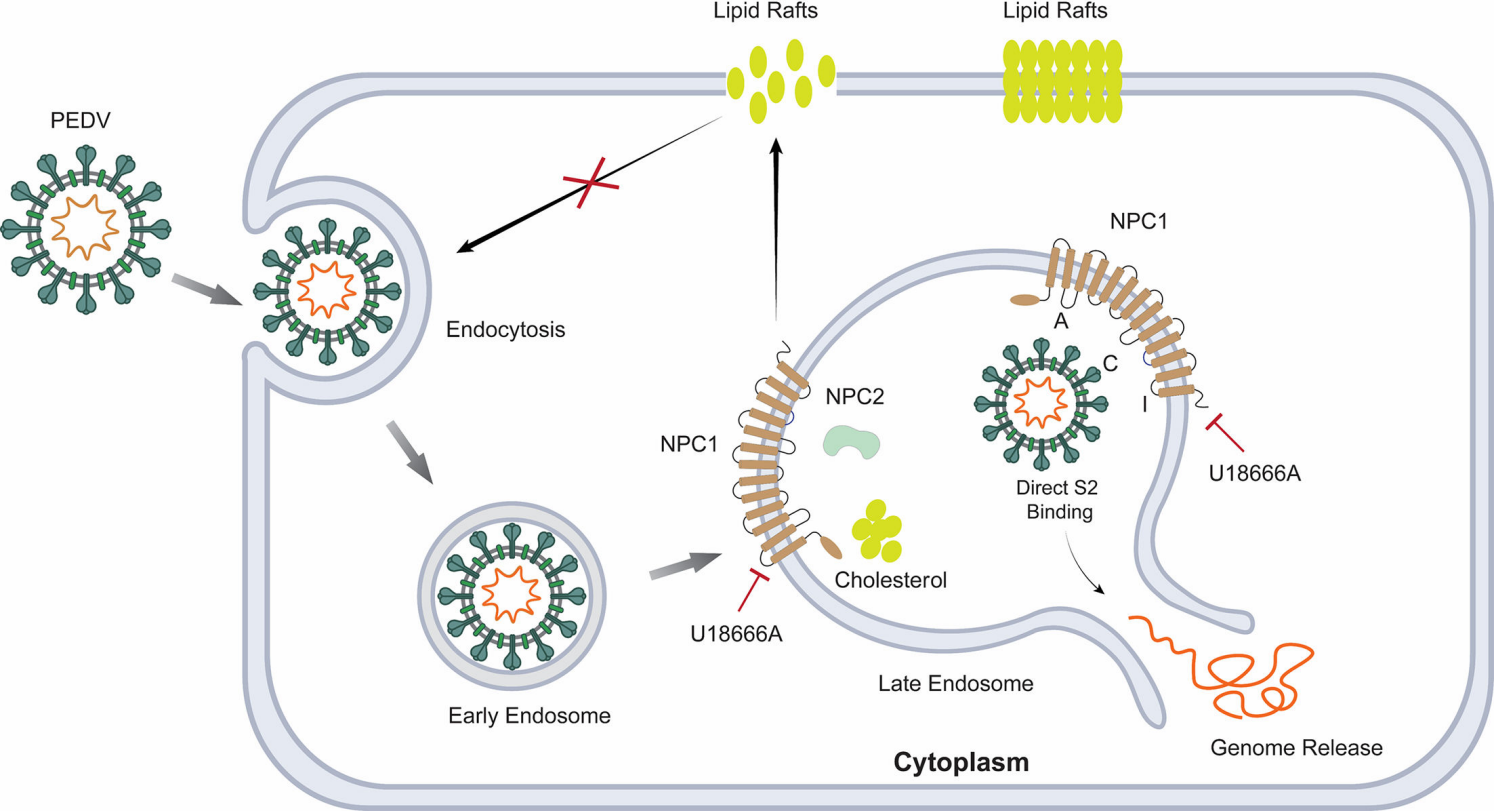
Proposed model of NPC1 role in PEDV invasion of host cells. NPC1 facilitates PEDV entry via two distinct pathways. First, the PEDV binds to cell surface receptors via its spike (S) protein and enters through endocytosis; this initial entry relies on NPC1-regulated plasma membrane cholesterol homeostasis and lipid raft structures. Following trafficking from early endosomes to late endosomes, the S2 subunit of PEDV S binds to NPC1-C on the late endosomal membrane. These interactions trigger fusion between the viral envelope and the endosomal membrane, releasing the viral genome into the cytoplasm. The inhibitor U18666A blocks PEDV invasion by disrupting NPC1-mediated cholesterol transport and interfering with the functional interaction between NPC1 and the viral S2 protein.

Integrating these insights, we propose a model for the role of NPC1 in PEDV entry (Fig. 11). After initial attachment and endocytosis, which depends on NPC1-maintained plasma membrane cholesterol, the virus traffics to late endosomes. Within this compartment, the proteolytically primed PEDV spike protein engages directly with NPC1 via its S2 subunit. This interaction acts as a critical trigger for the membrane fusion that releases the viral genome into the cytoplasm. The discovery that compounds like Tubeimosides block SARS-CoV-2 spike binding to NPC1-C provides a compelling precedent and indicates a specific molecular target for future antiviral development against PEDV (29). Our experimental data support this model. We first confirmed the direct interaction between the PEDV spike protein and NPC1 through molecular docking and co-immunoprecipitation. Functionally, the knockout of NPC1 significantly attenuated infection by both authentic PEDV and PEDV pseudoviruses, establishing its necessity for viral entry. While reintroduction of NPC1 into knockout cells increased PEDV infection, it did not restore it to wild-type levels. This incomplete rescue may reflect compensatory or adaptive changes that developed in the knockout clones during long-term NPC1 deficiency and were not readily reversed by NPC1 re-expression (53, 54). A key mechanistic insight came from overexpression studies in wild-type cells, which did not enhance PEDV infection, indicating that endogenous NPC1 levels are sufficient for entry, positioning it as a necessary but not rate-limiting factor. This saturation effect implies that infection efficiency is constrained by other processes, such as core receptor availability or protease activity. Furthermore, based on the established model of PEDV membrane fusion in late endosomes and lysosomes (11), the partial restoration of infection by exogenous cholesterol in U18666A-treated cells provides direct evidence for a dual role of NPC1: directly facilitating endosomal fusion and indirectly supporting viral entry through cholesterol maintenance.

Despite the substantial evidence, our study has several limitations. First, the mechanistic insights were primarily derived from human (Huh7) and monkey (Vero) cell lines. However, human and porcine NPC1 are highly conserved. Comparison of their amino acid sequences showed ~89.5% identity and 95.0% similarity across the full-length protein. In particular, the luminal domains A, C, and I analyzed in this study are well conserved, and several candidate residues implicated in our interaction analysis, including Y147, D228, N916, D917, Y932, and K1056, are identical between the two species (Fig. S3). Published cross-species analyses also support conservation of interaction-relevant regions within NPC1 domain C. Together, these observations strongly support the relevance of the mechanistic insights obtained in Huh7 cells to the porcine host, although minor species-specific differences cannot be fully excluded. Validation in primary porcine enterocytes, the natural target cell for PEDV, is essential to confirm the physiological relevance of these findings, as species-specific differences in cholesterol metabolism could exist. Second, the therapeutic potential of targeting NPC1 *in vivo* remains unexplored. Given NPC1’s vital role in cellular cholesterol homeostasis, systemic inhibition could pose toxicity risks. Future research should investigate localized delivery strategies to the intestinal epithelium to mitigate potential side effects.

In summary, we identified U18666A, a known NPC1 inhibitor, as a potent, broad-spectrum inhibitor of PEDV infection across multiple genotypes. This finding prompted an investigation of the relevant host determinants and revealed that NPC1 is essential for PEDV entry. NPC1 promotes viral internalization by directly interacting with the spike S2 subunit and by functionally cooperating with NPC2. By defining this host-virus interface, our work advances understanding of PEDV pathogenesis and identifies the NPC1 pathway as a promising target for broad-spectrum, host-directed antivirals.

## Data Availability

All data generated or analyzed during this study are included in this article. Additional raw data supporting this study’s conclusions are available from the corresponding author upon reasonable request.

## SUPPLEMENTAL MATERIAL

Fig. S1 jvi.00301-26-s0001.tif Validation of NPC1 knockout cell lines.

Fig. S2 jvi.00301-26-s0002.tif Binding affinity and binding mode of PEDV-S protein with NPC1.

Fig. S3 jvi.00301-26-s0003.tif High conservation of NPC1 between human and pig.

Supplemental material jvi.00301-26-s0004.docx Tables S1 and S2; legends for Fig. S1 to S3.

ASM does not own the copyrights to Supplemental Material that may be linked to, or accessed through, an article. The authors have granted ASM a non-exclusive, world-wide license to publish the Supplemental Material files. Please contact the corresponding author directly for reuse.
