# Supplementary figures and images for: The cholesterol transporter Niemann-Pick C1 facilitates the entry of porcine epidemic diarrhea coronavirus
Source: J Virol. 2026 Jun 9;100(7):e00301-26. doi: 10.1128/jvi.00301-26 (PMC13386893; doi:10.1128/jvi.00301-26)

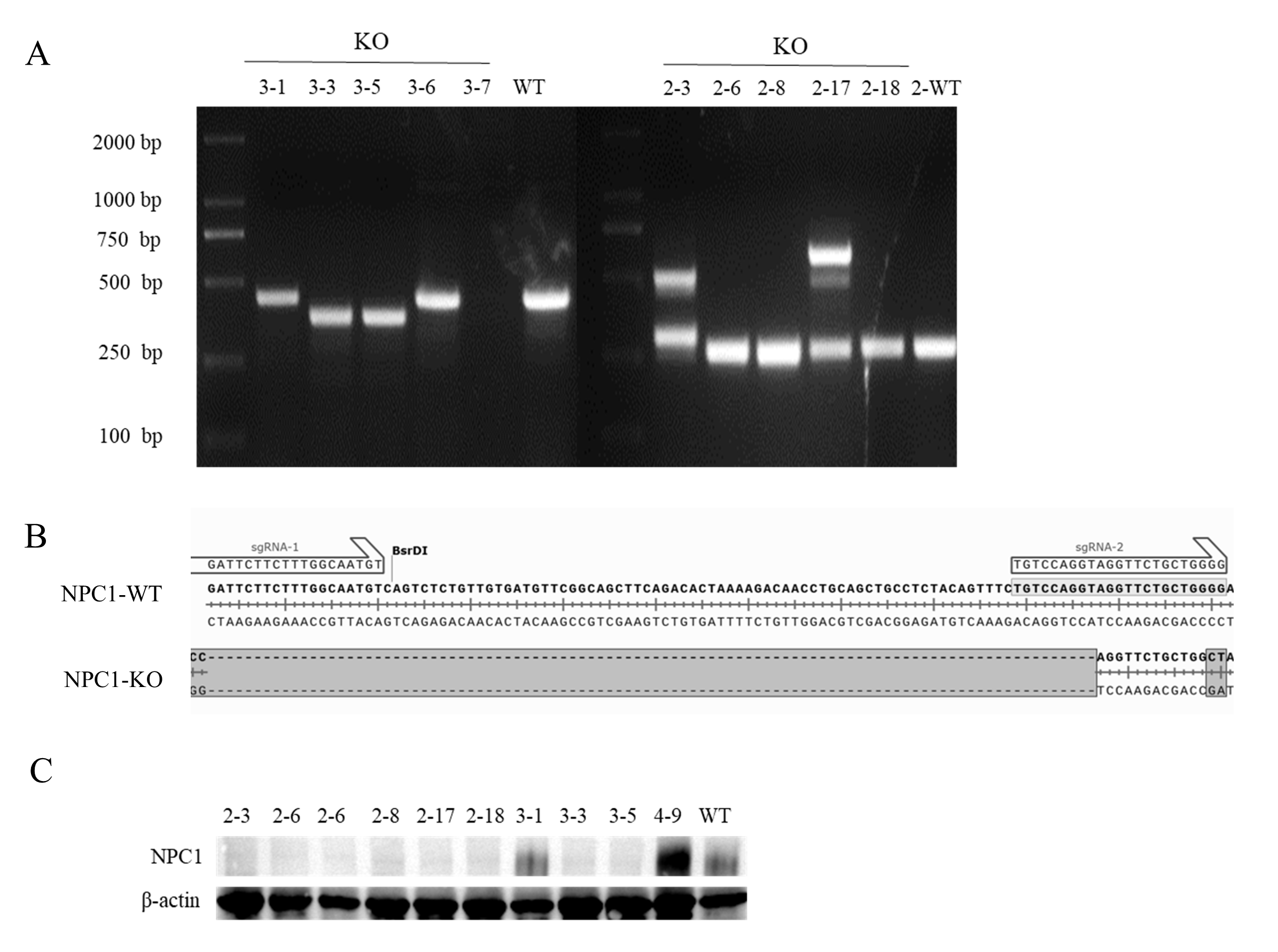

Supplement: Fig. S1 — Validation of NPC1 knockout cell lines. [file jvi.00301-26-s0001.tif]

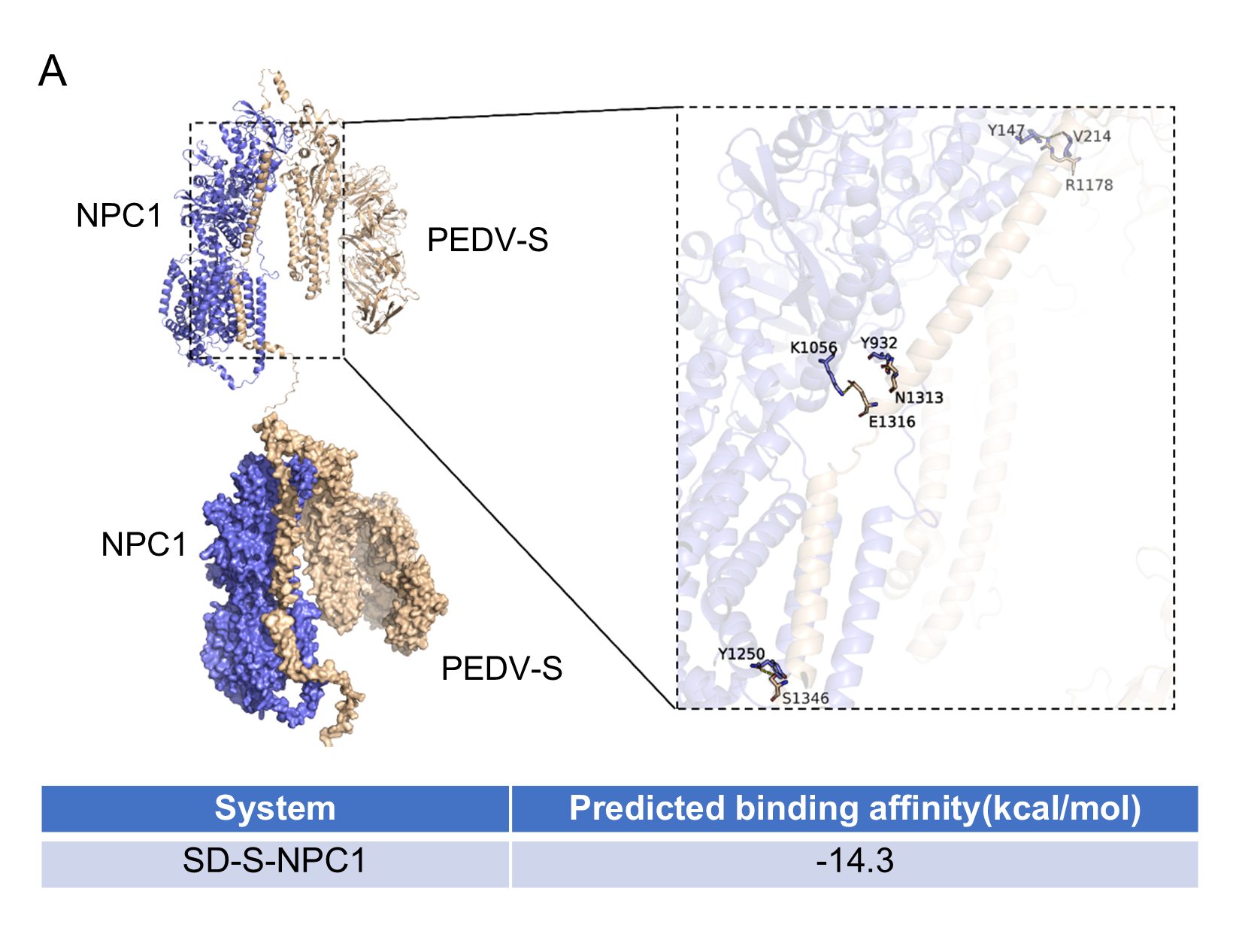

Supplement: Fig. S2 — Binding affinity and binding mode of PEDV-S protein with NPC1. [file jvi.00301-26-s0002.tif]

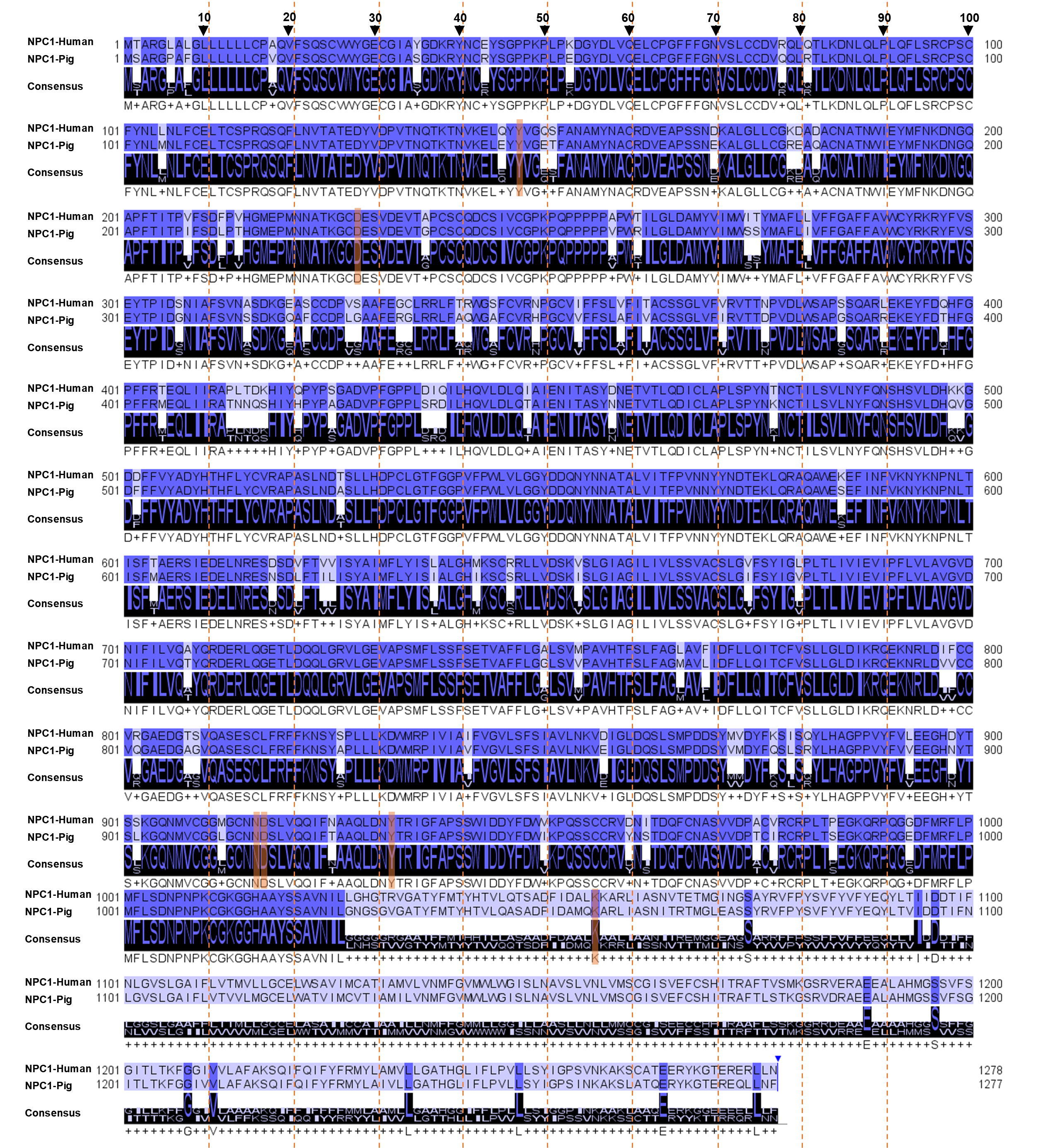

Supplement: Fig. S3 — High conservation of NPC1 between human and pig. [file jvi.00301-26-s0003.tif]
