# Supplementary material for: The cholesterol transporter Niemann-Pick C1 facilitates the entry of porcine epidemic diarrhea coronavirus
Source: J Virol. 2026 Jun 9;100(7):e00301-26. doi: 10.1128/jvi.00301-26 (PMC13386893; doi:10.1128/jvi.00301-26)
Supplement: Supplemental material — Tables S1 and S2; legends for Fig. S1 to S3. [file jvi.00301-26-s0004.docx]

Table S1. Oligo sequences used in this study

| Name^a^ | Sequence (5’-3’) | Usage |
| --- | --- | --- |
| PEDV-N-F | GAGGGTGTTTTCTGGGTTG | qPCR |
| PEDV-N-R | CGTGAAGTAGGAGGTGTGTTAG |  |
| PEDV-S-F | CTACTCCCGCCTCGACATTC |  |
| PEDV-S-R | TGAAGATATGCTCGCCGTCC |  |
| Human-Gadph-F | TGGTATCGTGGAAGGAC |  |
| Human-Gadph-R | GGATGACCTTGCCCACAGCC |  |
| Human-sgRNA-NPC1-1-F | CACCGACATTGCCAAAGAAGAATCC | konckout |
| Human-sgRNA-NPC1-1-R | AAACGGATTCTTCTTTGGCAATGTC |  |
| Human-sgRNA-NPC1-2-F | CACCGTGTCCAGGTAGGTTCTGCTG |  |
| Human-sgRNA-NPC1-2-R | AAACCAGCAGAACCTACCTGGACAC |  |
| Human-sgRNA-NPC1-3-F | CACCGAGACTCGTCACAGCCTTTGG |  |
| Human-sgRNA-NPC1-3-R | AAACCCAAAGGCTGTGACGAGTCTC |  |
| Human-sgRNA-NPC1-4-F | CACCGCCTGCTCCCTGGACGATCCT |  |
| Human-sgRNA-NPC1-4-R | AAACAGGATCGTCCAGGGAGCAGGC |  |
| Human-sgRNA-NPC2-1-F | CACCGTGGTGCTTTCCGGAGCCGGGGG |  |
| Human-sgRNA-NPC2-1-R | AAACACCGAAAGGCCTCGGCCCCC |  |
| Human-sgRNA-NPC2-2-F | CACCGCTGCCCAGGCCGAACCGGTG |  |
| Human-sgRNA-NPC2-2-R | AAACGACGGGTCCGGCTTGGCCAC |  |
| Human-sgRNA-NPC1-1KO-F | CAGGATGTGTCTTACCC | PCR |
| Human-sgRNA-NPC1-1KO-R | CGCATGCATACACATTATACATACA |  |
| Human-sgRNA-NPC1-2KO-F | CACAATTCCTTTCTGTAGATT |  |
| Human-sgRNA-NPC1-2KO-R | GTGCCAGTGGGCAATT |  |
| pcDNA3.1-NPC1-A-HA-F | ccactagtccagtgtggtggATGACAGCCAGAGGACTG |  |
| pcDNA3.1-NPC1-A-HA-R | aacgggccctctagactcgattaGGCGTAGTCAGGCACGTCGTATGGGTAAAAGGTCCACAGGATTGGTG |  |
| pcDNA3.1-NPC1-I-HA-F | ccactagtccagtgtggtggATGAAGGTGGACATTGGC |  |
| pcDNA3.1-NPC1-I-HA-R | aacgggccctctagactcgattaGGCGTAGTCAGGCACGTCGTATGGGTAGAAGATGGTGTCGTCGAT |  |
| pcDNA3.1-NPC1-C-HA-F | ccactagtccagtgtggtggATGTGGAGCGCCCCAAGC |  |
| pcDNA3.1-NPC1-C-HA-R | aacgggccctctagactcgattaGGCGTAGTCAGGCACGTCGTATGGGTAGACCACGGTGAACACGTCG |  |
| pcDNA3.1-S1-HA-F | ccactagtccagtgtggtggATGACCCCCCTGATC |  |
| pcDNA3.1-S1-HA-R | aacgggccctctagactcgattaGGCGTAGTCAGGCACGTCGTATGGGTACCTTGTGTTATTGAA |  |

Note: ^a^ F denotes forward PCR primer; R denotes reverse PCR primer. ^b^ homologous arms are lowercase.

Table S2. Dosages of cholesterol-related small molecules

| Compound | Concentration |
| --- | --- |
| U18666A | 1 μg/mL |
| LXR-623 | 5 μM |
| Atorvastatin | 10 μM |
| Evacetrapib | 3 μM |
| Dalcetrapib | 5 μM |
| Cholestyramine | 100 μM |
| Gemfibrozil | 100 μM |
| Fenofibrate | 20 μg/mL |
| Isoliquiritigenin | 5 μM |
| Simvastatin | 5 μM |

**Figure S1. Validation of NPC1 knockout cell lines.** **(A)** PCR analysis confirmed NPC1 knockout, with some clones showing a smaller product due to deletion and others showing no obvious size change. **(B)** Sanger sequencing confirmed the deletion in clones with the smaller PCR product. **(C)** Western blot analysis verified the absence of NPC1 protein in selected candidate clones, with β-actin as loading control.

Note: Panels A and C represent parallel validation of independent clones and are not in one-to-one correspondence. Clone 4-9 was included as an additional candidate for Western blotting but was not analyzed by PCR. Western blot was used as the final confirmation of protein knockout, as PCR alone cannot always reliably identify effective deletions.

**Figure S2. Binding affinity and binding mode of** **PEDV-S protein with NPC1.** The binding free energy of the PEDV‑S‑NPC1 complex is -14.3 kcal/mol. Negative values indicate favorable binding, with more negative values corresponding to stronger interactions. In the AlphaFold3‑predicted binding mode, PEDV‑S is shown in wheat, NPC1 is shown in blue, and hydrogen bonds are indicated by yellow dashed lines. Specifically, hydrogen bonds are formed between R1178, N1313, E1316, and S1346 of PEDV‑S and Y147, Y932, K1056, and Y1250 of NPC1. Hydrogen bonding is the predominant non‑covalent interaction governing the PEDV‑S‑NPC1 association. The extensive hydrogen bond network, together with the low binding energy, indicates a stable interaction between PEDV‑S and NPC1.

**Figure S3.** **High conservation of NPC1 between human and pig.** Amino acid sequence alignment of human and porcine NPC1 proteins shows 89.5% identity and 95.0% similarity across the full‑length sequences. Key residues identified as candidate interaction sites Y147, D228, N916, D917, Y932, and K1056 are fully conserved and highlighted with yellow boxes.
